# A Flexible Circularly Polarized Luminescence Switching Device Based on Proton‐Coupled Electron Transfer

**DOI:** 10.1002/advs.202202636

**Published:** 2022-07-21

**Authors:** Guojian Yang, Zhiqiang Yao, Xuefeng Yang, Yigui Xie, Pengfei Duan, Yu‐Mo Zhang, Sean Xiao‐An Zhang

**Affiliations:** ^1^ State Key Lab of Supramolecular Structure and Materials College of Chemistry Jilin University Changchun 130012 P. R. China; ^2^ CAS Center for Excellence in Nanoscience CAS Key Laboratory of Nanosystem and Hierarchical Fabrication National Center for Nanoscience and Technology (NCNST) Beijing 100190 P. R. China

**Keywords:** bistability, circularly polarized luminescences, electrically switchable devices, flexible devices

## Abstract

Flexible circularly polarized luminescence (CPL) switching devices have been long‐awaited due to their promising potential application in wearable optoelectronic devices. However, on account of the few materials and complicated design of manufacturing systems, how to fabricate a flexible electric‐field‐driven CPL‐switching device is still a serious challenge. Herein, a flexible device with multiple optical switching properties (CPL, circular dichroism (CD), fluorescence, color) is designed and prepared efficiently based on proton‐coupled electron transfer (PCET) mechanism by optimizing the chiral structure of switching molecule. More importantly, this device can maintain the switching performance even after 300 bending‐unbending cycles. It has a remarkable comprehensive performance containing bistable property, low open voltage, and good cycling stability. Then, prototype devices with designed patterns have been fabricated, which opens a new application pattern of CPL‐switching materials.

## Graphical abstract

A brand‐new optical switching system based on the PCET mechanism, which firstly integrates electrochemical information and multiple optical properties (CPL, CD, fluorescence and color) in a single photoelectric device efficiently.

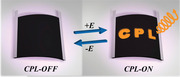

## 1 Introduction

Switchable circularly polarized luminescence (CPL) boosts the development of luminescence materials with chiral property^[^^]^ in optoelectronic devices,^[^ ^4^, ^5^, ^6^ ^]^ information encryption,^[^ ^7^, ^8^ ^]^ efficient biological probes,^[^ ^9^, ^10^ ^]^ etc. Several stimuli‐responsive CPL materials, driven by various kinds of stimulation containing pH,^[^ ^11^, ^12^, ^13^, ^14^ ^]^ light,^[^ ^15^, ^16^, ^17^ ^]^ electric field (e‐field),^[^ ^18^, ^19^ ^]^ solvent,^[^ ^20^, ^21^ ^]^ and ionic additives,^[^ ^22^, ^23^, ^24^ ^]^ have been developed, exhibiting attractive and promising properties. Among those materials, electrically switchable CPL materials, which can be integrated into photoelectric devices to enable them easy to be controlled by the electrical signals, are regarded as one of the most important approaches for achieving the practical application of CPL materials. For instance, chiral liquid crystal devices with the ability of emitting CPL under a suitable e‐field (CP‐LCDs) have been demonstrated by doping the chiral compounds into the liquid crystal.^[^ ^25^, ^26^ ^]^ However, the previous CP‐LCDs are all prepared based on rigid substrates, due to the intrinsic fluidity of liquid crystal materials. This insurmountable barrier has undoubtedly restricted the further application of electrically switchable CPL materials in the promising field of smart wearable electronics, which should be constructed with flexible substrates and materials.

Fabricating a solid or semi‐solid electro‐responsive CPL‐switchable film is the first step to achieve desirable flexible devices. Electrochromic (EC) materials with outstanding features of easy preparation of semi‐solid film, good structural modifiability, and low working voltage have the potential to make a breakthrough in this field.^[^ ^27^, ^28^, ^29^ ^]^ The research of EC materials grafting chiral groups is becoming a rising hotspot.^[^ ^30^, ^31^, ^32^ ^]^ For instance, a multiple chiral switching device with rich optical properties inheriting from base‐responsive dyes has been fabricated based on proton‐coupled electron transfer (PCET) mechanism.^[^ ^30^ ^]^ In addition, some chiroptical molecular switches have been realized by the combination of chiral chromophores with electrochemically active viologen derivatives^[^ ^33^ ^]^ or some conducting polymers.^[^ ^34^ ^]^ Generally, the obtained EC materials exhibited the differential absorption of left‐ and right‐handed circularly polarized light (CD, circular dichroism), rather than the differential emission. This was owing to their weak CD signals or poor emission ability. Therefore, how to fabricate a flexible CPL‐switching device based on EC materials remains a serious challenge.

In this work, a flexible electrically switchable CPL device was designed and fabricated based on the PCET mechanism by combining a newly developed acid‐responsive CPL‐switching material (R‐Rhodol‐A, 6'‐(diethylamino)‐3‐oxo‐4’‐((E)‐(((R)‐1‐phenylethyl)iminio)methyl)‐3H‐ spiro[isobenzofuran‐1,9’‐xanthen]‐3’‐olate) and an “electroacid” (Urea‐N, 1‐(4‐(dimethyl‐amino)phenyl)‐3‐(p‐tolyl)urea). This device exhibited a CPL‐switching ability as it could be reversibly switched between CPL‐silent state (“OFF”) and emitting a left‐handed orange‐yellow CPL state (“ON”) with the regulation of electricity, accompanied by an apparent change of color (colorless to pink). In addition, the flexible device exhibits dually stable optical states (“OFF” and “ON”) with a promising comprehensive performance containing long maintaining time of “ON” state (>3600 s) without power supply, low open voltage (+0.7 V) and good cycling stability (>1600 cycles). More importantly, the flexible device can maintain the switching performance even after 300 bending‐unbending cycles.

## 2 Results and Discussion

### 2.1 Screening of the Acid‐Responsive CPL Switching Molecule

**Figure 1:**
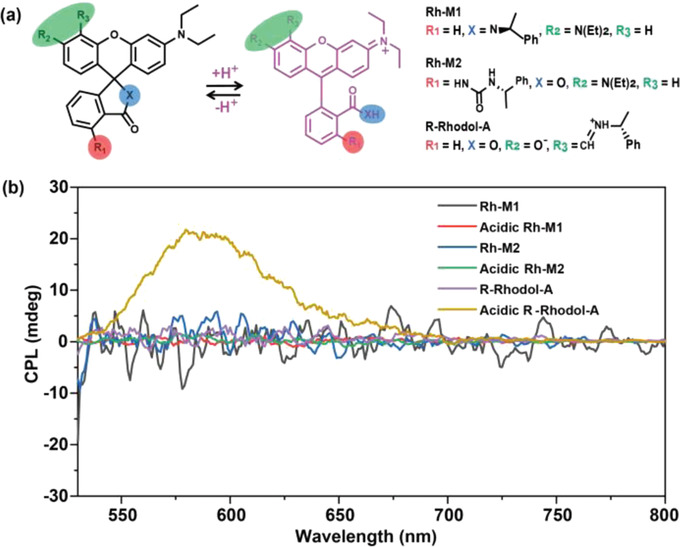
a) Design of the acid‐responsive CPL switching molecules with various chiral sites. b) CPL spectra of the PMMA films doped with *Rh‐M1*, *Rh‐M2*, *R‐Rhodol‐A*, and their acidic structural forms, respectively (*λ*_ex_ = 410 nm).

In order to develop an anticipated electrically switchable CPL device, the designed functional materials should contain both the CPL activity and electro‐responsive property. Based on the PCET mechanism in our previous work,^[^ ^28^, ^29^, ^30^ ^]^ the rich optical properties of acid‐responsive materials could be regulated by the electro‐redox of some functional molecules/structures (“electroacid”: the proton can be released reversibly during the electrochemical process). Therefore, designing a high‐performance acid‐responsive molecule with promising CPL properties is the key to fabricate an ideal CPL‐switching device. Based on this principal, three fluorescent dyes with a sensitive acid‐responsive functional group and a suitable chiral‐leader have been designed and synthesized (**Figure** **1**a and Figures S19–S25, Supporting Information). The chiral benzylamine was introduced into the different sites of rhodamine/rhodol (Rh‐M1, Rh‐M2, and R‐Rhodol‐A). Then, their acid‐responsive spectral properties were tested. Compared with the other two molecules, R‐Rhodol‐A showed the most promising acid‐responsive chiroptical switching property (Figure 1b) based on its obvious acid‐responsive CPL activity when doped into the polymethyl methacrylate (PMMA) film. Its switchable CPL property may be due to its high‐efficiency chirality transfer from chiral center to the fluorophore based on intramolecular hydrogen bond (Figures S1a and S25, Supporting Information). Besides, R‐Rhodol‐A showed a more sensitive acid‐responsive photoluminescence (PL) ability compared to the other two molecules (Figure S1b, Supporting Information). According to the above experimental results, R‐Rhodol‐A was chosen as the acid‐responsive CPL‐switching material to be combined with an “electroacid,” for constructing a new electrically switchable CPL system.

### 2.2 Feasibility of the E‐Field‐Driven CPL‐Switching Property

**Figure 2:**
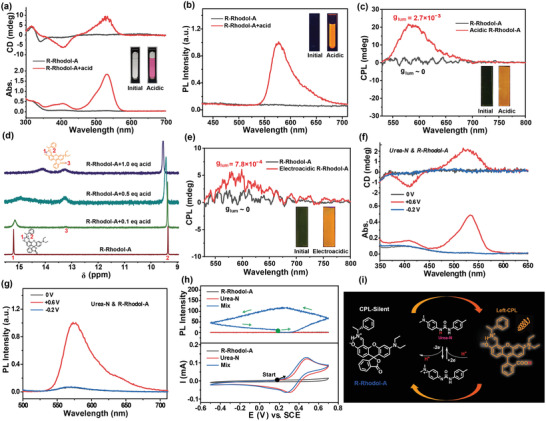
a) CD, UV–vis absorption, and b) PL spectra (*λ*_ex_ = 365 nm) of 2.0 × 10^–4^ m *R‐Rhodol‐A* when added 0.0 and 1.0 eq CF_3_COOH (acid) in acetonitrile, respectively (*d* = 2 mm). c) CPL spectra (*λ*_ex_ = 410 nm) of the PMMA films doped with *R‐Rhodol‐A* and acidic *R‐Rhodol‐A*, respectively. d) ^1^H NMR spectra of 1.7 × 10^–2^ m *R‐Rhodol‐A* when added various eq CF_3_COOH in deuterated DMSO recorded at 400 MHz. e) CPL spectra of the PMMA films doped with *R‐Rhodol‐A, Urea‐N*, and TBAPF_6_ before and after added suitable voltage, respectively (*λ*_ex_ = 410 nm). f) CD and UV–vis absorption spectra, and g) PL spectra (*λ*_ex_ = 365 nm) of the mixture (mix) of 2.0 × 10^–4^ m *R‐Rhodol‐A*, 1.0 × 10^–3^ m *Urea‐N* in acetonitrile with 1.0 × 10^–1^ m TBAPF_6_, when added various voltages, respectively (*d* = 1 mm), h) Changes in fluorescence of 574 nm (top) and cyclic voltammograms (CVs, bottom) in situ of 2.0 × 10^–4^ m *R‐Rhodol‐A*, 1.0 × 10^–3^ m *Urea‐N*, and the mix of 2.0 × 10^–4^ m *R‐Rhodol‐A* and 1.0 × 10^–3^ m *Urea‐N* in acetonitrile with 1.0 × 10^–1^ m TBAPF_6_. i) Schematic diagram of the CPL‐switching mechanism induced by *Urea‐N* as the “electroacid.”

Firstly, the acid‐responsive chiroptical properties of R‐Rhodol‐A were thoroughly measured both in solution and film. In a dilute solution (acetonitrile as the solvent), CD signal showed apparent changes along with the appearance of strong orange‐yellow fluorescence and color (colorless to pink), when the acidity of this system was increased (**Figure** **2**a,b and Figure S2, Supporting Information). However, it was difficult to obtain an apparent CPL signal when R‐Rhodol‐A was dispersed in a dilute solution (Figure S3, Supporting Information). Fortunately, promising acid‐responsive CPL‐switching properties could be observed when R‐Rhodol‐A was doped into transparent PMMA films (Figures S4 and S5, Supporting Information). As shown in Figure 2c, this functional film was CPL‐silent (*g*_lum_ ≈ 0) in visible band at the initial state, while the acidified film could emit a left‐handed orange‐yellow CPL (*λ*_em_ = 580 nm, *g*_lum_ ≈ 2.7 × 10^–3^) under a suitable excitation light (*λ*_ex_ = 410 nm). As expected, the acidified PMMA film doped with S‐Rhodol‐A showed a symmetrical CPL‐emitting property (Figure S6, Supporting Information). Then, the structural changes of R‐Rhodol‐A during its acid‐responsive process were tested by ^1^H nuclear magnetic resonance (^1^H NMR) spectroscopy (Figure 2d and Figure S27, Supporting Information). With the increment of chemical acid in the system, the No. 3 hydrogen (H) appeared and increased, accompanied by the change of chemical shifts of No. 1 H and No. 2 H. Compared with the acid‐response structural switching of Rhodol,^[^ ^35^ ^]^ the acid‐responsive mechanism of R‐Rhodol‐A was demonstrated in Figure S7 (Supporting Information). This unique acid‐responsive CPL‐switchable property laid the basis for preparation of electrically switchable thin‐film devices based on “electroacid” method and PCET mechanism.

Further, to switch the CPL state of R‐Rhodol‐A under the stimulation of electricity, Urea‐N was used as the “electroacid” due to its promising ability of reversible proton‐release.^[^ ^23^ ^]^ Functional PMMA films doped with mixed systems of Urea‐N and R‐Rhodol‐A were prepared, respectively (Figure 2e). This film exhibited CPL‐silent state (*g*_lum_ ≈ 0) in visible band without the stimulation of electricity. After adding +1.2 V bias voltage, a left‐handed orange‐yellow CPL appeared (*λ*_em_ = 580 nm, *g*_lum_ ≈ 7.8 × 10^–4^). This trend kept the same/similar with the acidic process of PMMA doped with the R‐Rhodol‐A. Additionally, when the functional molecule changed from R‐Rhodol‐A to S‐Rhodol‐A, the film showed a symmetrical CPL‐switching property (Figure S8, Supporting Information). The above results indicated that the electrically switchable CPL could be achieved by combining the CPL molecular switch (R‐Rhodol‐A/S‐Rhodol‐A) and an “electoacid” (Urea‐N).

To investigate the switching mechanism, electro‐redox behaviors of the above two components were tested firstly (Figure S9, Supporting Information). The oxidation potential peak of Urea‐N was +0.50 V, while R‐Rhodol‐A was +1.00 V. This big potential gap ensured that R‐Rhodol‐A would not be oxidized/reduced when Urea‐N was oxidized/reduced. Further, in situ chiroptical changes of mixed and individual solutions of Urea‐N and R‐Rhodol‐A under the electricity were measured to investigate the electrical responsive ability. As revealed in Figure 2f,g, the new CD signal of mixture at 532 nm could be observed under +0.6 V, accompanied by the emergence of a new absorbance peak at 532 nm and a new emission peak at 574 nm (excited by 365 nm). Those new optical peaks could be eliminated reversibly by the stimulation of −0.2 V. However, no spectral change of individual solutions was observed under the same stimulation (Figure S10, Supporting Information). In addition, the intensity of fluorescence and absorption could be changed reversibly with the oxidation/reduction of Urea‐N, only when Urea‐N and R‐Rhodol‐A coexisted in a system (Figure 2h and Figure S11, Supporting Information). It meant that the mixture solution of Urea‐N and R‐Rhodol‐A exhibited the anticipated switchable optical property depended on the electro‐redox of Urea‐N. More importantly, the switched spectra of CD signal, color and fluorescence regulated by the electricity kept the same/similar with that of R‐Rhodol‐A treated by chemical acid. Therefore, the reversible optical change of R‐Rhodol‐A is due to the reversible release of proton of Urea‐N under the stimulation of e‐field.

According to the above experimental results and PCET mechanism,^[^ ^28^, ^29^, ^30^ ^]^ we can verify the CPL‐switching property of *R‐Rhodol‐A* controlled by the electro‐redox of *Urea‐N* (Figure 2i). At the initial neutral system, *R‐Rhodol‐A* with a ring‐closed state was CPL‐silent. When the system was added a suitable positive voltage, oxidization of *Urea‐N* will release a proton to generate a strong acidity. At the same time, the surrounding *R‐Rhodol‐A* can capture the released proton, generating the ring‐open pattern of this acid‐responsive molecule. This process can induce a left‐handed orange‐yellow CPL emission. More importantly, this system could be integrated into PMMA films easily, which was beneficial for the fabrication of flexible devices.

### 2.3 Fabrication of the E‐Field‐Driven CPL‐Switching Devices

**Figure 3:**
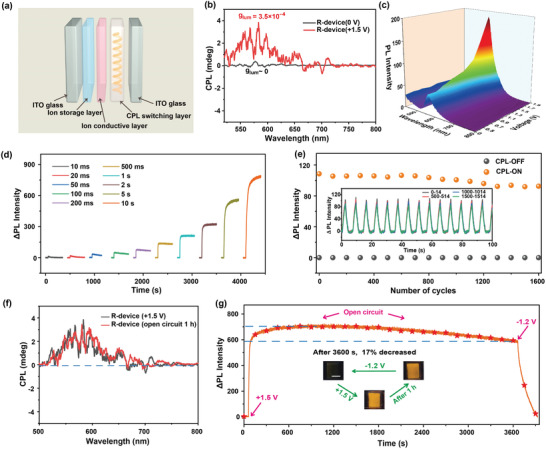
a) Structure of the e‐field‐driven CPL‐switching device. b) CPL spectra of the R‐device before and after added suitable voltage (+1.5 V) (*λ*_ex_ = 410 nm). c) PL spectra of the device under various positive voltages (*λ*_ex_ = 410 nm). d) The changes of PL intensity at 574 nm of the device stimulated with +1.5 V for different times (*λ*_ex_ = 410 nm). e) Cycling stability of PL intensity of the device under a suitable e‐field (+1.5 V for CPL‐ON, −1.2 V for CPL‐OFF, *λ*_ex_ = 410 nm). f) CPL spectra of the bistable R‐device under stimulation of +1.5 V and its corresponding spectra after power off 3600 s, respectively. g) PL intensity of the device at 574 nm under stimulation of +1.5 V for CPL‐ON, power off 3600 s, −1.2 V for CPL‐OFF. Inset: pictures of the bistable device (scale bar: 1 cm).

To promote the application of this CPL‐switching system, we have fabricated an electrically switchable device with five functional layers (two indium tin oxide (ITO) glass electrodes, a CPL‐switching layer, an ion conductive layer and an ion storage layer) (**Figure** **3**a). The fabrication parameters (thickness of CPL‐switching layer, molar ratio of R‐Rhodol‐A to Urea‐N) were optimized by considering the contrast ratio of PL intensity of devices (Figure S12, Supporting Information). Fortunately, this device exhibited a unique CPL‐switching ability (Figure 3b). At the initial state, the device displayed a CPL‐silent state (*g*_lum_ ≈ 0) in visible band. After adding a suitable oxidation potential (+1.5 V), this device emitted a left‐handed orange‐yellow CPL (*λ*_em_ = 580 nm, *g*_lum_ ≈ 3.5 × 10^–4^). In addition, the CPL could be eliminated reversibly with a suitable opposed voltage (−1.2 V), which meant the switching process was reversible completely. As shown in Figure S13 (Supporting Information), this device could emit a right‐handed CPL under the electricity when the functional molecule in CPL‐switching layer changed from R‐Rhodol‐A (R‐device) to S‐Rhodol‐A (S‐device). In addition, the luminescence intensity showed an obvious voltage‐dependence property (Figure 3c and Figure S14, Supporting Information). The optical change can be observed when the stimulated voltage is above +0.7 V. This low threshold voltage benefited from the low oxidation potential of Urea‐N, as well as the PCET mechanism. Moreover, the change of luminescence intensity could also be adjusted by the stimulation time, the longer the stimulation time, the greater the change (Figure 3d). Interestingly, the change of PL intensity could be detected when the short switching time was more than 10 ms at +1.5 V. Cycling stability was also a necessary metric when evaluating the performance of an electrically switchable optical device. As shown in Figure 3e and Figure S15 (Supporting Information), the optical switching performance (fluorescence and color) of this device showed no apparent attenuation after 1600 “OFF‐ON” cycles. More importantly, the CPL‐ON state could maintain the emitting state over 1 h at an open circuit state without the power supply (Figure 3f and Figure S16, Supporting Information), which meant that the switchable CPL device exhibited a good bistable property. To study the bistability more clearly, the detailed changes of luminescence intensity were investigated by in situ PL spectra (Figure 3g). The fluorescence intensity of this device degraded about 17% after 3600 s. Based on the above results, an electrically switchable bistable CPL device with promising performances was fabricated successfully.

### 2.4 Applications of the E‐Field‐Driven CPL‐Switching Device

**Figure 4:**
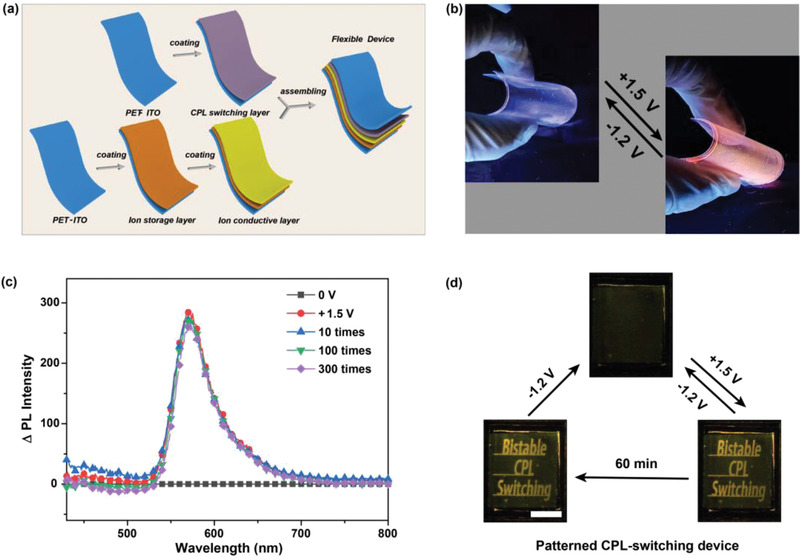
a) Preparation process and b) physical display (emission pattern, under a UV light at 365 nm) of the flexible device, respectively. c) PL spectra of the flexible device on different bending times (*λ*_ex_ = 410 nm). d) Bistable CPL‐switching process of the patterned device (+1.5 V for CPL‐ON, then power off 3600 s, −1.2 V for CPL‐OFF). (Scale bar: 1 cm).

Due to the flexibility of CPL‐switching layer and the other two functional layers, the bendable device could be fabricated based on polyethylene terephthalate (PET)‐ITO electrodes (**Figure** **4**a). The prepared device could regulate CPL states reversibly even at a highly bent state, as shown in Figure 4b and Figure S17 (Supporting Information) (radius of curvature was 1.2 cm). In addition, this device could maintain the switching performance even after 300 bending‐unbending cycles (Figure 4c), indicating the high durability. According to the above results, this newly developed CPL‐switching system had a potential application in future wearable electronics. Then, to explore other application scenarios of this CPL‐switching device, we have made a patterned device and observed its bistable CPL‐switching performance (Figure 4d, Figure S18 and Video S1, Supporting Information). At the initial state, the device was colorless and non‐luminous. Then, the information of “Bistable CPL Switching” was displayed with both color and emission (under a UV light at 365 nm) when added a suitable stimulation voltage (+1.5 V). The appeared information could maintain above 3600 s after removing the voltage. Then, the information could be erased completely by adding an opposite voltage (−1.2 V). This unique bistable information‐switching ability is suitable for the energy‐saving CPL‐switching devices and encryption devices. Compared with the CPL switching devices produced from other materials, the prepared device based on PCET mechanism exhibited promising performances on flexibility, bistability and cycling stability, which is very important for the real application of CPL‐switching materials (Figure S28, Supporting Information). However, the value of *g*_lum_ was too small. Thus, how to improve the value of glum is the key point in the further research.

## 3 Conclusions

In summary, we have developed a brand‐new optical switching system based on the PCET mechanism, which firstly integrated electrochemical information and multiple optical properties (CPL, CD, fluorescence, and color) in a single photoelectric device efficiently. The prepared device showed a unique electrically switchable CPL property with outstanding overall performances as short response time (<100 ms), low open voltage (+0.7 V), long cycling stability (>1600 cycles) and good bistability (>3600 s). More importantly, flexible devices were constructed by the combination of bendable CPL‐switching materials and PET‐ITO substrates, which showed promising switching properties and ideal durability. This work provides a creative strategy for fabricating smart flexible CPL‐switching devices, which will undoubtedly promote the applications of CPL‐switching materials.

## Conflict of Interest

The authors declare no conflict of interest.

## Supporting information

Supporting Information

Supplemental Video 1

## Data Availability

The data that support the findings of this study are available in the supplementary material of this article.
