# Supplementary material for: A Flexible Circularly Polarized Luminescence Switching Device Based on Proton‐Coupled Electron Transfer
Source: Adv Sci (Weinh). 2022 Jul 21;9(26):2202636. doi: 10.1002/advs.202202636 (PMC9475559; doi:10.1002/advs.202202636)
Supplement: Supplementary file 1 — Supporting Information [file ADVS-9-2202636-s001.pdf]

ADVANCED SCIENCE
Open Access

Supporting Information

for *Adv. Sci.*, DOI 10.1002/advs.202202636

A Flexible Circularly Polarized Luminescence Switching Device Based on Proton-Coupled Electron Transfer

Guojian Yang, Zhiqiang Yao, Xuefeng Yang, Yigui Xie, Pengfei Duan, Yu-Mo Zhang* and Sean Xiao-An Zhang*

Supporting information

A Flexible Circularly Polarized Luminescence Switching Device Based on Proton-Coupled Electron Transfer

Guojian Yang, Zhiqiang Yao, Xuefeng Yang, Yigui Xie, Pengfei Duan, Yu-Mo Zhang*, and Sean Xiao-An Zhang*

G. Yang, Z. Yao, Y. Xie, Y.-M. Zhang, S. X.-A. Zhang
State Key Lab of Supramolecular Structure and Materials, College of Chemistry, Jilin University, Changchun, 130012, China
E-mail: zhangyumo@jlu.edu.cn

X. Yang, P. Duan
CAS Center for Excellence in Nanoscience, CAS Key Laboratory of Nanosystem and Hierarchical Fabrication, National Center for Nanoscience and Technology (NCNST), Beijing, 100190, China.
E-mail: duanpf@nanoctr.cn

Table of contents

1. General experimental details..3
2. Acid-responsive properties of the chiroptical switches........................10
3. Spectroelectrochemical measurements *in situ*.......................................18
4. Supplementary Figures for devices..21
5. ^{1}H NMR and ^{13}C NMR spectra...28
6. Comparison and References..37

1. General experimental details

Materials

Trifluoroacetic acid (CF_3COOH, TFA), 2-(4-diethylamino-2-hydroxybenzoyl)benzoic acid, resorcinol, triethylamine (Et_3N, TEA), p-benzoquinone (p-BQ), sodium tert-butoxide (t-BuONa), hydroquinone (HQ), tetrabutyl ammonium hydroxide (TBAOH), N, N-dimethylbenzene-1,4-diamine, 1-isocyanato-4-methylbenzene, (S)-(-)-α-Methylbenzyl isocyanate and tetrabutylammonium hexafluorophosphate ($TBAPF_6$) were purchased from Energy Chemicals, China. Propylene carbonate (PC), (R)-(+)-α-Methylbenzylamine were bought from Adamas-beta®, China. Rhodamine B, phosphorus oxychloride, 1,2-dichloroethane, acetyl chloride, Polymethyl methacrylate (PMMA), ferrocene were purchased from Aladdin Chemicals, China. Hexamethylenetetramine was purchased from Tianjin Beilian Fine Chemicals Development Co., Ltd. Indium tin oxide (ITO) electrodes were purchased from South China Xiang Science & Technology company. All the solvents were purchased in commercialized way and used without further purification. $TBAPF_6$ was recrystallized for three times in anhydrous ethanol and dried under vacuum overnight at 80 ^oC before using in the electrochemical measurements.

Characterizations

UV-Vis absorption spectra and kinetic data of absorption intensity were measured using a Shimadzu UV-2550 PC double-beam spectrophotometer. Electrochemical data were measured by a Bio-logic electrochemical work station. Fluorescence emission spectra and kinetic data of fluorescence intensity were measured by a Shimadzu spectrofluorimeter RF-5301PC. Circular dichroism (CD) spectra and the corresponding extinction spectra were collected on a Chiralscan Plus V100 circular dichroism spectrometer (Applied Photophysics Ltd.). ^1H, ^{13}C and ^1H Cosy NMR spectra were measured by a ZhongkeNiujin AS 400 M NMR spectrometer. Chemical shift values were given relative to tetramethylsilane (TMS). LC-HRMS analysis was obtained by an Agilent 1290-microTOF-Q II mass spectrometer. Melting points were determined using the SGW X-4B microscopy melting point apparatus (Shanghai). Indium tin oxide (ITO) electrodes were etched by the Xi-Ai FB30-Z HPWU0300-SKS Laser marking machine. Circularly polarized luminescence (CPL) spectra were measured by a JASCO CPL-200 and a CPL-300 spectrophotometer and the excitation wavelength was set at 410 nm.

Synthesis of (S)-3',6'-bis(diethylamino)-2-(1-phenylethyl)spiro[isoindoline-1,9'-xanthen]-3-one molecule (Rh-M1)

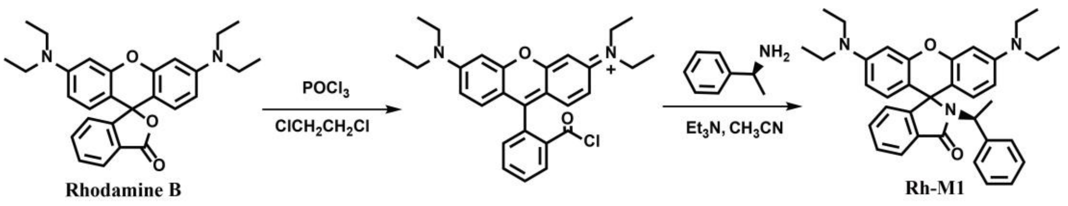

Scheme S1. Synthetic route of the molecule *Rh-M1*.

2.40 g (5.00 mmol) Rhodamine B was dissolved in 40 mL 1,2-dichloroethane, then 3.75 mL (40.00 mmol) phosphorus oxychloride was slowly added into the solution. The mixture was heated to reflux for 3 hours. Then, the solvent of the reaction mixture was distilled off to obtain violet oil. The residue was dissolved in 50 mL acetontrile, then 1.03 mL (8.00 mmol) (S)-(-)-α-Methylbenzylamine and 5.00 mL (36.00 mmol) triethylamine was added into the mixture. The system was stirred for 3 hours at room temperature. After the reaction finished, solvent was evaporated under vacuum. The crude product was obtained after dissolving in dichloromethane (CH_2Cl_2), washing with the deionized water, drying with anhydrous sodium sulfate (Na_2SO_4) and evaporated. Then, the crude product was purified on a silica gel column eluting with ethyl acetate (EtOAc):petroleum ether=1:10 to obtain the final pink product *Rh-M1*. The yield was 40%. The characterization was shown below (**Figure S19**). ^{1}H NMR (400 MHz, DMSO) δ 7.77-7.70 (m, 1H), 7.55-7.46 (m, 2H), 7.07-6.98 (m, 4H), 6.94 (d, *J*=6.1 Hz, 2H), 6.39 (d, *J*=10.6 Hz, 4H), 6.11-5.99 (m, 2H), 4.08 (q, *J*=7.0 Hz, 1H), 3.42-3.21 (m, 8H), 1.42 (d, *J*=7.2 Hz, 3H), 1.08 (dt, *J*=16.7, 6.9 Hz, 12H). ^{13}C NMR (101 MHz, DMSO) δ 166.51 (s), 153.51 (s), 153.36 (s), 152.89 (s), 148.93 (s), 148.78 (s), 142.24 (s), 133.04 (s), 132.60 (s), 129.92 (s), 129.30 (s), 128.81 (s), 127.91 (s), 127.82 (s), 126.83 (s), 124.23 (s), 122.51 (s), 108.53 (s), 105.45 (s), 105.39 (s), 97.66 (s), 97.59 (s), 65.88 (s), 53.03 (s), 44.29 (s), 44.19 (s), 18.97 (s), 12.94 (s), 12.79 (s). HRMS: m/z = 546.3114 $[M+H]^+$, calculated 546.3115.The melting point is 177 ^oC-179 ^oC.

Synthesis of (S)-1-(3',6'-bis(diethylamino)-3-oxo-3H-spiro[isobenzofuran-1,9'-xanthen]-4-yl)-3-(1-phenylethyl)urea molecule (Rh-M2)

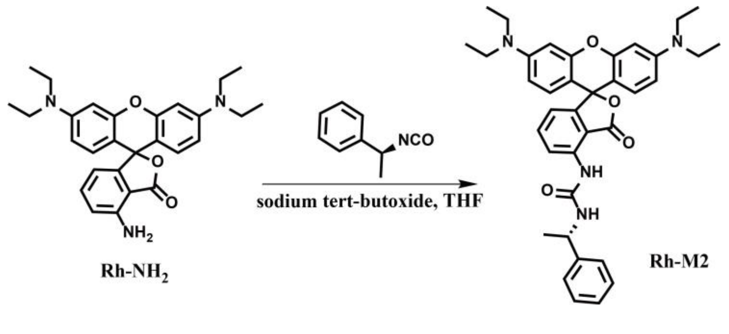

Scheme S2. Synthetic route of the molecule *Rh-M2*.

Detailed synthetic process of *Rh-NH₂* was reported in our previous work (*Nat. Mater.* 2019, 18, 1335-1342). 281.68 μL (2.00 mmol) (S)-(-)-α-Methylbenzyl isocyanate was dissolved in 10 mL tetrahydrofuran (THF), then the solution was slowly added into the mixture of 457.57 mg (1.00 mmol) *Rh-NH₂* and 192.20 mg (2.00 mmol) sodium tert-butoxide (t-BuONa) in 20 mL THF at 0 °C. The system was stirred for 3 hours at room temperature. After the reaction finished, solvent was evaporated under vacuum. The crude product was obtained after dissolving in dichloromethane (CH_2Cl_2), washing with the deionized water, drying with anhydrous sodium sulfate (Na_2SO_4) and evaporated. Then, the crude product was purified on a silica gel column eluting with methanol (CH_3OH):dichloromethane (CH_2Cl_2)=1:50 to obtain final pink red product *Rh-M2*. The yield was 50%. The characterization was shown below (**Figure S20**). ^{1}H NMR (400 MHz, DMSO) δ 9.07 (s, 1H), 8.34 (t, *J*=9.3 Hz, 2H), 7.57 (t, *J*=8.0 Hz, 1H), 7.37 (d, *J*=7.7 Hz, 4H), 7.26 (d, *J*=6.6 Hz, 1H), 6.65 (d, *J*=7.5 Hz, 1H), 6.54 (d, *J*=8.7 Hz, 2H), 6.43 (d, *J*=8.6 Hz, 4H), 4.88 (t, *J*=7.3 Hz, 1H), 3.40-3.31 (m, 8H), 1.42 (d, *J*=6.9 Hz, 3H), 1.09 (td, *J*=7.0, 3.4 Hz, 12H). ^{13}C NMR (101 MHz, DMSO) δ 170.10 (s), 154.16 (s), 153.33 (s), 153.08 (s), 149.62 (s), 145.61 (s), 140.04 (s), 137.08 (s), 129.16 (s), 128.82 (s), 127.19 (s), 126.45 (s), 117.60 (s), 115.89 (s), 112.11 (s), 108.69 (s), 105.70 (s), 97.42 (s), 85.38 (s), 49.45 (s), 44.30 (s), 23.39 (s), 12.85 (s). HRMS: m/z = 605.3119 $[M+H]^+$, calculated 605.3122. The melting point is 147 °C-149 °C.

Synthesis of 6'-(diethylamino)-3-oxo-4'-((E)-(((R)-1-phenylethyl)iminio)methyl)-3H-spiro[isobenzofuran-1,9'-xanthen]-3'-olate (R-Rhodol-A) and 6'-(diethylamino)-3-oxo-4'-((E)-(((S)-1-phenylethyl)iminio)methyl)-3H-spiro[isobenzofuran-1,9'-xanthen]-3'-olate (S-Rhodol-A)

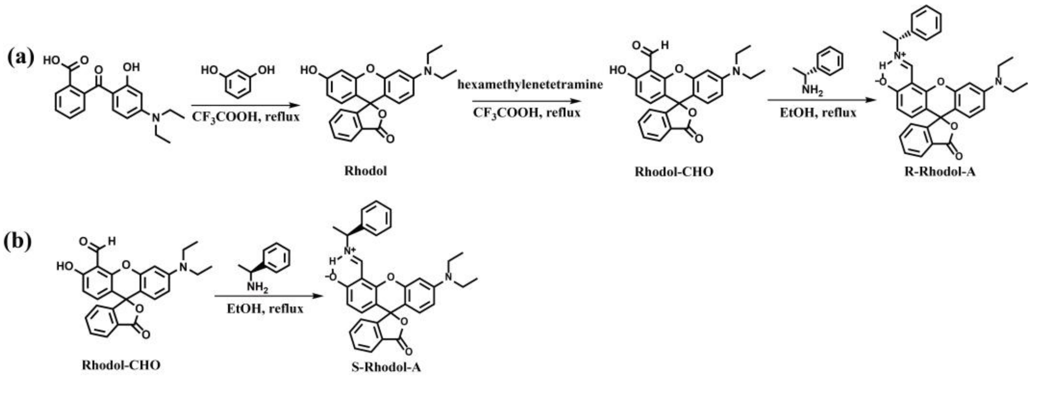

Scheme S3 Synthetic route of the molecule (a) *R-Rhodol-A*, and (b) *S-Rhodol-A*, respectively.

(1) Synthesis of *Rhodol* molecule. Detailed synthetic process of *Rhodol* was reported in our previous work (*Chem. Commun.*, 2017, 53, 11209-11212). The characterization was shown below (**Figure S21**). ^{1}H NMR (400 MHz, DMSO) δ 10.07 (s, 1H), 7.98 (d, J=7.5 Hz, 1H), 7.78 (t, J=7.3 Hz, 1H), 7.70 (t, J=7.4 Hz, 1H), 7.27 (d, J=7.6 Hz, 1H), 6.67 (s, 1H), 6.53 (s, 2H), 6.45 (d, J=4.6 Hz, 3H), 3.40-3.33 (m, 4H), 1.09 (t, J=6.9 Hz, 6H). LC-HRMS: m/z = 388.1539 $[M+H]^+$, calculated 388.1543.

(2) Synthesis of *Rhodol-CHO* molecule. 387.50 mg (1.00 mmol) *Rhodol* and 211.2 mg hexamethylenetetramine (1.50 mmol) were dissolved in 20 mL trifluoroacetic acid (CF_3COOH) and the mixture was heated to reflux for 12 hours. Then, 3 mL deionized water was added into the system and the system kept stirring at 95 °C for 2 hours. After the reaction finished, solvent was evaporated under vacuum. The crude product was obtained after dissolving in dichloromethane (CH_2Cl_2), washing with the saturated sodium bicarbonate ($NaHCO_3$) aqueous solution, drying with anhydrous sodium sulfate (Na_2SO_4) and evaporated. Then, the crude product was purified on a silica gel column eluting with methanol (CH_3OH):dichloromethane (CH_2Cl_2)=1:70 to obtain final pink product *Rhodol-CHO*. The yield was 40%. The characterization was shown below (**Figure S22**). ^{1}H NMR (400 MHz, DMSO) δ 11.89 (s, 1H), 10.68 (s, 1H), 8.01 (d, J=7.4 Hz, 1H), 7.81 (t, J=7.3 Hz, 1H), 7.73 (t, J=7.3 Hz, 1H), 7.32 (d, J=7.5 Hz, 1H), 6.93 (d, J=8.8 Hz, 1H), 6.69 (d, J=7.4 Hz, 2H), 6.50 (s, 2H), 3.37 (d, J=6.7 Hz, 4H), 1.11 (t, J=6.5 Hz, 6H). ^{13}C NMR (101 MHz, DMSO) δ 193.74 (s), 169.13 (s), 163.32 (s), 153.34 (s), 152.59 (s), 151.88 (s), 149.81 (s), 137.11 (s), 136.16 (s), 130.70 (s), 129.00 (s), 126.74 (s), 125.18 (s), 124.53 (s), 113.50 (s), 110.53 (s), 109.76 (s), 109.60 (s), 104.72 (s), 97.83 (s), 83.12 (s), 44.27 (s), 12.84 (s). HRMS: m/z = 416.1488 $[M+H]^+$, calculated 416.1492. The melting point is 212 °C-214 °C.

(3) Synthesis of *R-Rhodol-A* molecule. 415.50 mg (1.00 mmol) *Rhodol-CHO* and 141.80 μL (R)-(+)-α-Methylbenzylamine (1.10 mmol) were dissolved in 20 mL anhydrous ethyl alcohol (C_2H_5OH) and the mixture was heated to reflux for 12 hours. After the reaction finished, solvent was evaporated under vacuum. Then, the crude product was purified on a silica gel column eluting with methanol (CH_3OH):dichloromethane (CH_2Cl_2)=1:50 to obtain the final pale pink product *R-Rhodol-A*. The yield was 40%. The characterization was shown below (**Figure S23**). ^{1}H NMR (400 MHz, DMSO) δ 15.19 (s, 1H), 9.35 (s, 1H), 7.99 (d, J=7.5 Hz, 1H), 7.80 (t, J=7.4 Hz, 1H), 7.71 (t, J=7.4 Hz, 1H), 7.47 (d, J=7.4 Hz, 2H), 7.42 (t, J=7.3 Hz, 2H), 7.33 (d, J=7.0 Hz, 1H), 7.29 (t, J=6.7 Hz, 1H), 6.63 (d, J=8.1 Hz, 2H), 6.54-6.44 (m, 3H), 4.99 (d, J=6.2 Hz, 1H), 3.37 (d, J=6.9 Hz, 4H), 1.65 (d, J=6.4 Hz, 3H), 1.12 (t, J=6.6 Hz, 6H). ^{13}C NMR (101 MHz, DMSO) δ 169.26 (s), 167.64 (s), 159.86 (s), 152.83 (s), 152.10 (s), 151.38 (s), 149.70 (s), 143.61 (s), 136.04 (s), 133.23 (s), 130.54 (s), 129.25 (s), 129.02 (s), 127.92 (s), 126.90 (s), 126.79 (s), 125.06 (s), 124.49 (s), 115.29 (s), 109.52 (s), 106.99 (s), 105.69 (s), 105.07 (s), 97.65 (s), 84.04 (s), 65.55 (s), 44.23 (s), 24.52 (s), 12.91 (s). HRMS: m/z = 519.2273 $[M+H]^+$, calculated 519.2278. The melting point is 235 °C-237 °C.

(4) Synthesis of *S-Rhodol-A* molecule. 415.50 mg (1.00 mmol) *Rhodol-CHO* and 141.80 μL (S)-(-)-α-Methylbenzylamine (1.10 mmol) were dissolved in 20 mL anhydrous ethyl alcohol (C_2H_5OH) and the mixture was heated to reflux for 12 hours. After the reaction finished, solvent was evaporated under vacuum. Then, the crude product was purified on a silica gel column eluting with methanol (CH_3OH):dichloromethane (CH_2Cl_2)=1:50 to obtain the final pale pink product *S-Rhodol-A*. The yield was 40%. The characterization was shown below (**Figure S24**). ^{1}H NMR (400 MHz, DMSO) δ 15.20 (s, 1H), 9.35 (s, 1H), 7.99 (d, J=7.4 Hz, 1H), 7.80 (t, J=7.5 Hz, 1H), 7.71 (t, J=7.3

Hz, 1H), 7.47 (d, J=7.1 Hz, 2H), 7.42 (t, J=7.2 Hz, 2H), 7.33 (d, J=7.0 Hz, 1H), 7.28 (d, J=7.6 Hz, 1H), 6.63 (d, J=7.7 Hz, 2H), 6.54-6.45 (m, 3H), 5.06-4.93 (m, 1H), 3.37 (d, J=6.7 Hz, 4H), 1.65 (d, J=6.2 Hz, 3H), 1.12 (t, J=6.5 Hz, 6H). ^{13}C NMR (101 MHz, DMSO) δ 169.26 (s), 167.66 (s), 159.87 (s), 152.84 (s), 152.09 (s), 151.37 (s), 149.69 (s), 143.60 (s), 136.04 (s), 133.24 (s), 130.54 (s), 129.25 (s), 129.03 (s), 127.93 (s), 126.89 (s) 126.80 (s), 125.06 (s), 124.50 (s), 115.30 (s), 109.52 (s), 106.98 (s), 105.68 (s), 105.06 (s), 97.64 (s), 84.04 (s), 65.53 (s), 44.23 (s), 24.52 (s), 12.91 (s). HRMS: m/z = 519.2273 $[M+H]^+$, calculated 519.2278. The melting point is 235 ^{o}C-237 ^{o}C.

Preparation of the acid-responsive films

Herein, PMMA was used as the film-forming material and *R-Rhodol-A* was used as the acid responsive molecule. And, the doping content of *R-Rhodol-A* in PMMA was optimized after considering the PL intensity (**Figure S4**), which was 2 mg *R-Rhodol-A* doped in 100 mg PMMA. Thus, the detailed film-forming process could be described as below: (1) 2 mg *R-Rhodol-A* and 100 mg PMMA were dissolved in 1 mL CH_3CN. (2) The solution was blade coated on a transparent ITO glass to form an acid responsive transparent film. (Thickness was 20 μm)

Synthesis of 1-(4-(dimethyl-amino)phenyl)-3-(p-tolyl)urea (Urea-N)

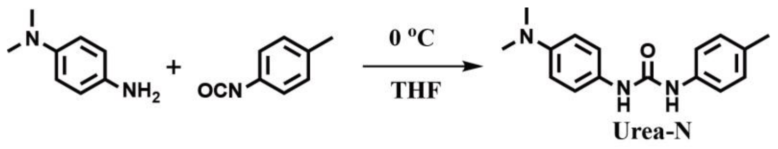

Scheme S4. Synthetic route of the molecule *Urea-N*.

Detailed synthetic process of *Urea-N* was reported in our previous work (*Nat. Commun.*, 2019, 10, 1559). The characterization was shown below (**Figure S26**). ^{1}H NMR (400 MHz, DMSO) δ 8.40 (s, 1H), 8.24 (s, 1H), 7.31 (d, J=8.4 Hz, 2H), 7.24 (d, J=9.0 Hz, 2H), 7.06 (d, J=8.3 Hz, 2H), 6.69 (d, J=9.0 Hz, 2H), 2.82 (s, 6H), 2.23 (s, 3H).

Electrochemistry

Cyclic voltammetry (CV) analysis was measured using a three-electrode system in acetonitrile (chromatographic grade) containing $TBAPF_6$ (0.1 mol/L) as the supporting electrolyte. The three-electrode system contained a glass-carbon working electrode (3 mm diameters, Chenhua, China), a Pt wire counter electrode (ida, China) and an Ag wire reference electrode (ida, China). The surface of the working electrode was polished with 0.3 and 0.05 μm alumina (ida, China) followed by ultrasonic cleaning in deionized water for three times.

Measurement methods of spectroelectrochemistry 'in situ' in solutions

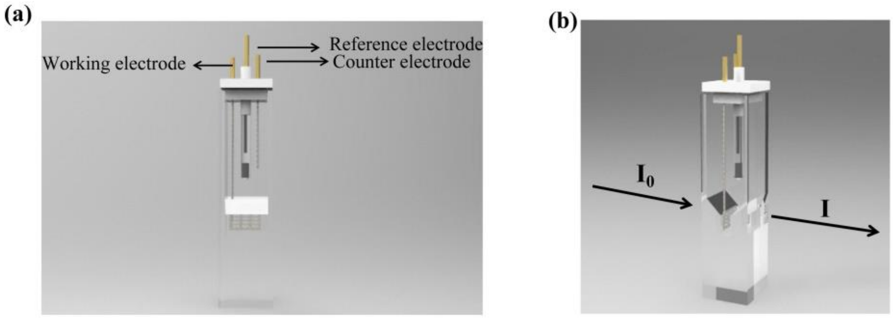

Scheme S5. (a) The structure of a thin-layer quartz electrochemical cell which was used to measure the redox states in solution '*in situ*'. (b) The transmission route of ultraviolet (UV) and visible (Vis) lights.

As shown in **Scheme S5**, the measurements of spectroelectrochemistry '*in situ*' were performed in a thin-layer (1 mm) quartz glass electrochemical cell (ida, China). The three electrodes contained a Pt network working electrode, a Pt wire counter electrode, and an Ag reference electrode.

Fabrication of the devices based on ITO glasses

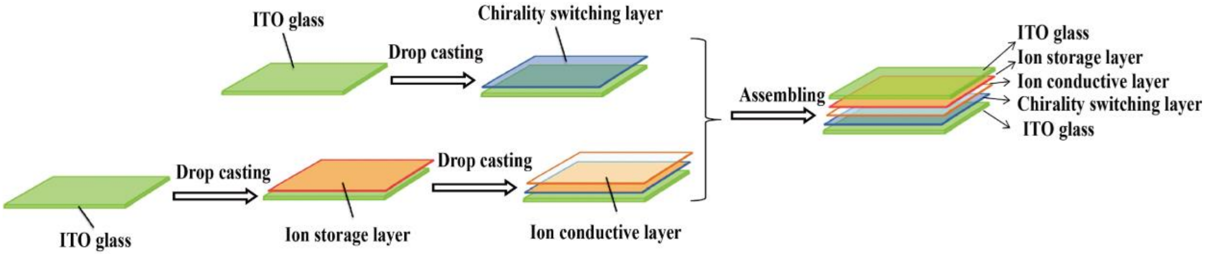

Scheme S6. Fabrication process of e-field-driven bistable CPL-switching device based on ITO glasses electrodes.

Firstly, functional solutions were prepared as below. (1) Chirality switching solution: A mixture of PMMA (1.00 g), $TBAPF_6$ (417 mg), PC (0.624 mL), Urea-N (2.25 mg) and R-Rhodol-A (20.0 mg) was dissolved in 10 mL CH_3CN. (2) Ion conductive solution: A mixture of PMMA (3.60 g), $TBAPF_6$ (1.50 g) and PC (0.75 mL) was dissolved in 20 mL CH_3CN. (3) Ion storage solution: A mixture of PMMA (0.72 g), $TBAPF_6$ (0.30 g), PC (0.15 mL) was dissolved in 10 mL CH_3CN with p-BQ (5.0×10^{-2} mol/L) and HQ (1.0×10^{-1} mol/L). The above solutions were heated at 40 ^{o}C and stirred until the solutions turned transparent.

After the preparation of functional solutions, the detailed fabrication process of devices was shown in **Scheme S6**. Chirality switching layer was formed by drop casting (thickness: 23 μm) on an ITO glass as the working electrode. And, ion storage layer was formed by drop casting on another ITO glass as the counter electrode. Then, ion conductive layer was coated above the ion

storage layer by drop casting. Finally, two electrodes with functional layers were connected tightly and the device was assembled successfully.

Fabrication of the devices based on PET-ITO electrodes

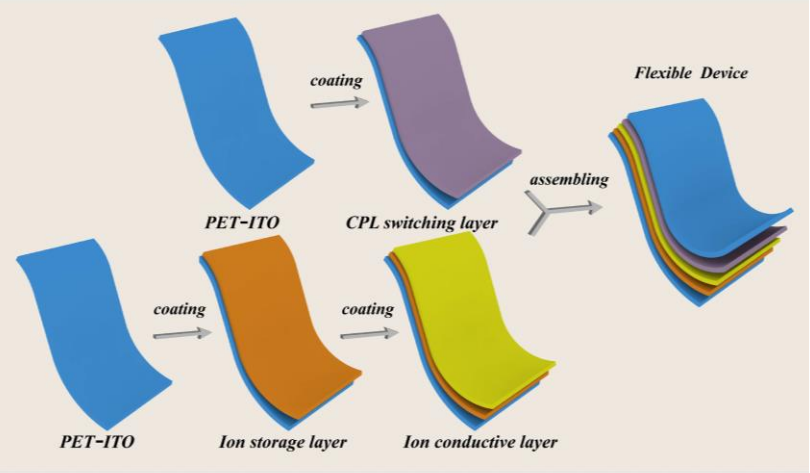

Scheme S7. Fabrication process of e-field-driven bistable CPL-switching device based on PET-ITO electrodes.

Firstly, functional solutions were prepared as below. (1) Chirality switching solution: A mixture of PMMA (1.00 g), $TBAPF_6$ (417 mg), PC (0.624 mL), Urea-N (2.25 mg) and R-Rhodol-A (20.0 mg) was dissolved in 10 mL CH_3CN. (2) Ion conductive solution: A mixture of PMMA (3.60 g), $TBAPF_6$ (1.50 g) and PC (0.75 mL) was dissolved in 20 mL CH_3CN. (3) Ion storage solution: A mixture of PMMA (0.72 g), $TBAPF_6$ (0.30 g), PC (0.15 mL) was dissolved in 10 mL CH_3CN with p-BQ (5.0×10^{-2} mol/L) and HQ (1.0×10^{-1} mol/L). The above solutions were heated at 40 ^{o}C and stirred until the solutions turned transparent.

After the preparation of functional solutions, the detailed fabrication process of devices was as shown in **Scheme S7**. Chirality switching layer was formed by drop casting (thickness: 23 μm) on a PET-ITO as the working electrode. And, ion storage layer was formed by drop casting on another PET-ITO as the counter electrode. Then, ion conductive layer was coated above the ion storage layer by drop casting. Finally, two electrodes with functional layers were connected tightly and the device was assembled successfully.

2. Acid-responsive properties of the chiroptical switches

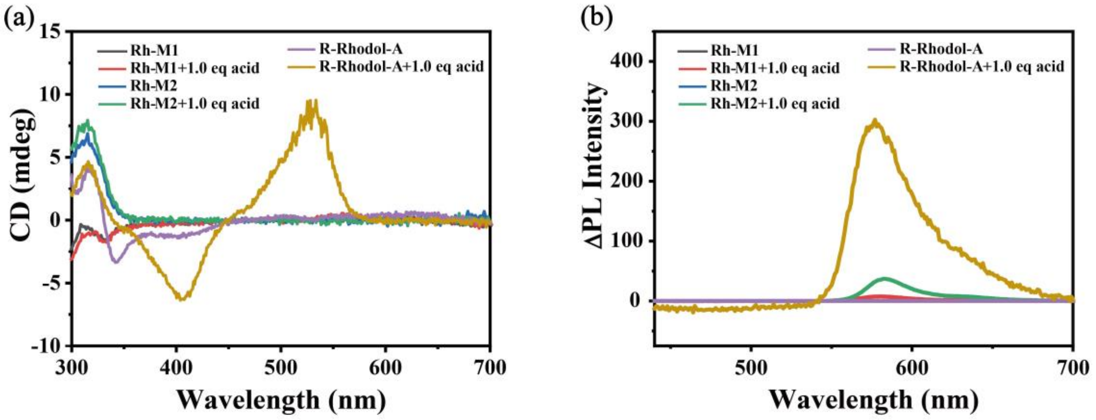

Figure S1. (a) CD spectra, and (b) fluorescence emission spectra (slit: 1.5, 3; ex: 365 nm) of the 2.0×10^{-4} M *Rh-M1*, *Rh-M2* and *R-Rhodol-A* when added 0.0 eq and 1.0 eq CF_3COOH (acid) in acetonitrile, respectively. (d=2 mm)

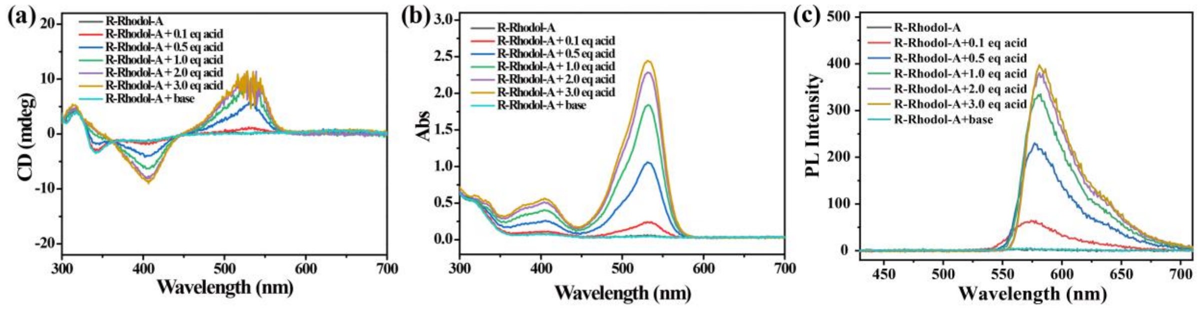

Figure S2. Acid-responsive properties of *R-Rhodol-A* in solution. (a) CD, (b) UV-Vis absorption spectra, (c) fluorescence emission spectra (slit: 1.5, 3; ex: 365 nm) of 2.0×10^{-4} mol/L *R-Rhodol-A* in acetonitrile (CH_3CN) when added 0.0, 0.1, 0.5, 1.0, 2.0, 3.0 equivalents (eq) chemical acid (CF_3COOH) and chemical base (TBAOH) (d=2 mm), respectively.

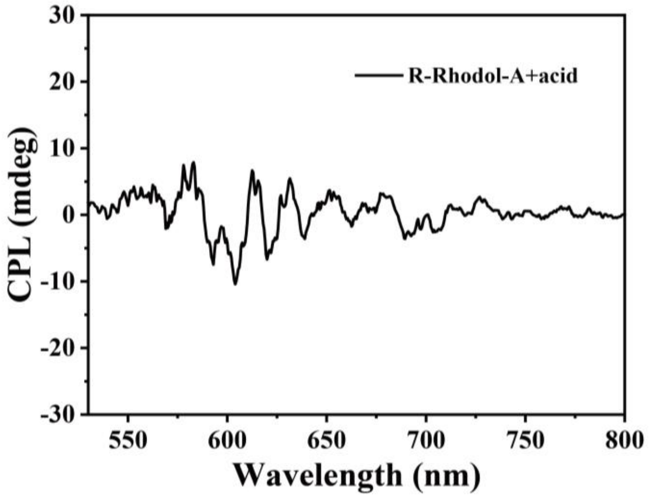

Figure S3. CPL spectra of 2.0×10^{-4} mol/L *R-Rhodol-A* in acetonitrile (CH_3CN) when added 1.0 equivalent (eq) chemical acid (CF_3COOH) (d=2 mm).

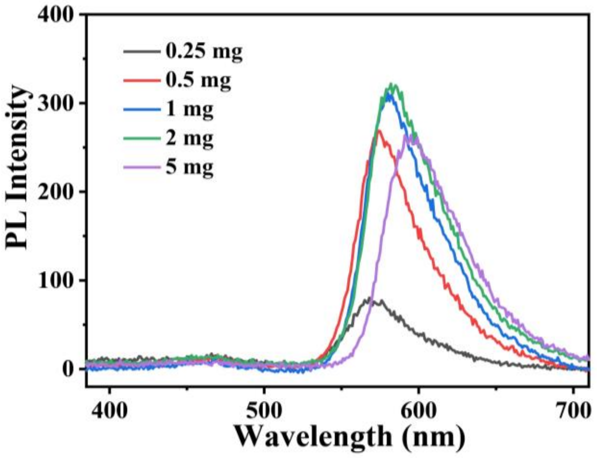

Figure S4. Optimization of the doped amount of *R-Rhodol-A* in PMMA. Fluorescence emission spectra of the films prepared by *R-Rhodol-A* with different amounts doped into 100 mg PMMA. (slit: 3, 5; ex: 365 nm)

Discussion: As shown in **Figure S4**, with the increment of doped amount of *R-Rhodol-A* in PMMA from 0.25 mg to 2 mg, the emission intensity increased apparently. However, when the doped mass increased to 5 mg, the emission intensity was decreased which was caused by the aggregation induced quenching (ACQ) effect. Thus, after considering the emission intensity of CPL, the doped amount of the functional acid-responsive CPL-switching film was selected as 2 mg *R-Rhodol-A* in 100 mg PMMA.

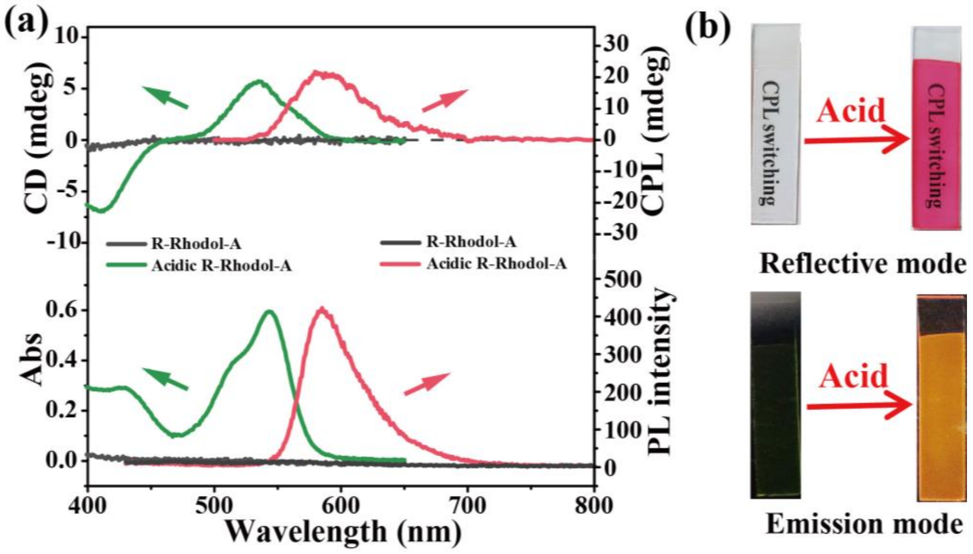

Figure S5. Acid-responsive property of *R-Rhodol-A* doped in PMMA film. (a) CD, CPL, UV-Vis absorption and fluorescence emission spectra of *R-Rhodol-A* and acidic *R-Rhodol-A*. (slit: 3, 5; ex: 410 nm) (b) Physical display of the functional PMMA film before and after added acid on the reflective mode and emissive mode (under irradiation of UV light (365 nm))

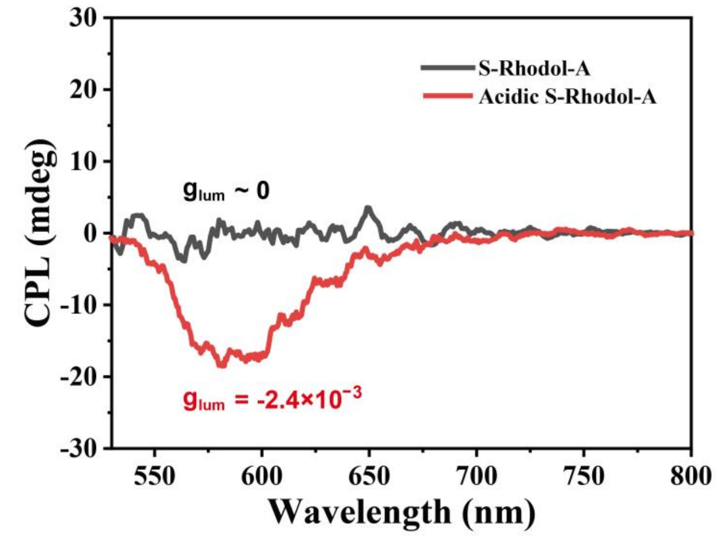

Figure S6. CPL spectra (λ_{ex}-410 nm) of the PMMA films doped with *S-Rhodol-A* and acidic *S-Rhodol-A*, respectively.

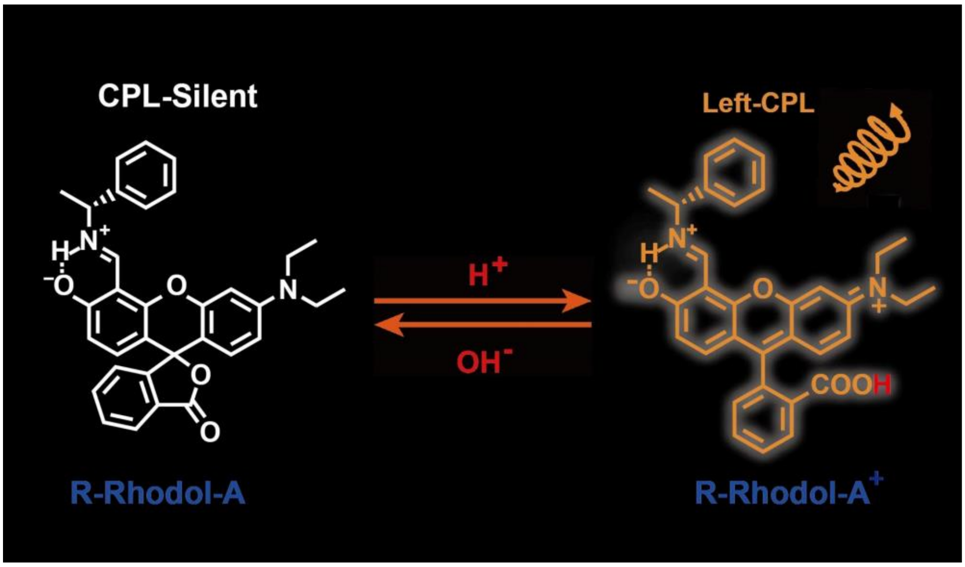

Figure S7. Schematic diagram of the acid-responsive CPL-switching mechanism of *R-Rhodol-A*.

Discussion: With the addition of acid, the *R-Rhodol-A* was changed from ring-closed state to ring-open state, accompanied the switching from CPL-silence state to emitting left orange-yellow CPL state.

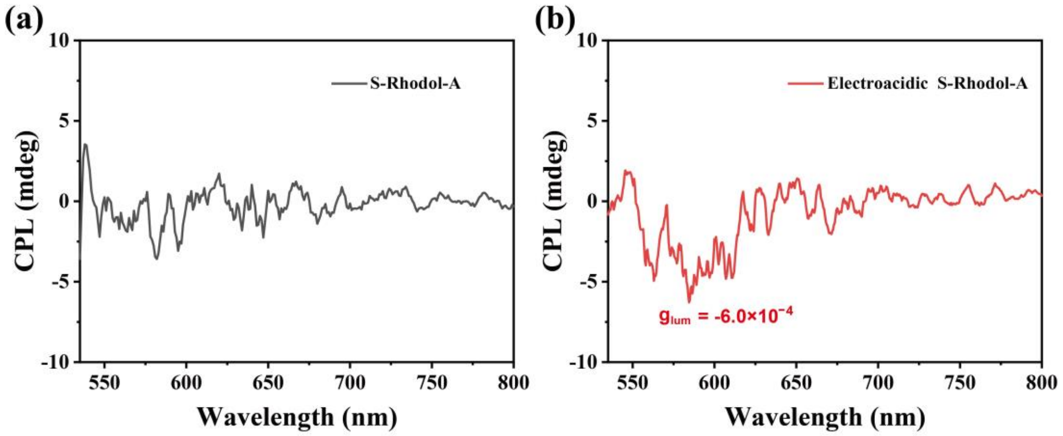

Figure S8. CPL spectra of the PMMA films doped with *S-Rhodol-A*, *Urea-N* and $TBAPF_6$ before and after added suitable voltage, respectively (λ_{ex}=410 nm).

3. Spectroelectrochemical measurements *in situ*

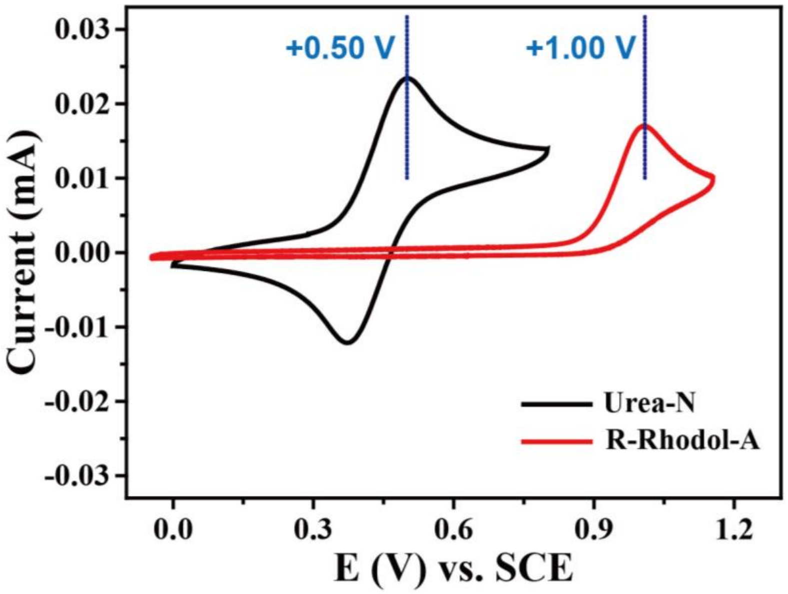

Figure S9. CV curves of the individual solutions of 1.0×10^{-3} mol/L Urea-N, 1.0×10^{-3} mol/L *R-Rhodol-A* with 1.0×10^{-1} mol/L $TBAPF_6$ in CH_3CN, respectively.

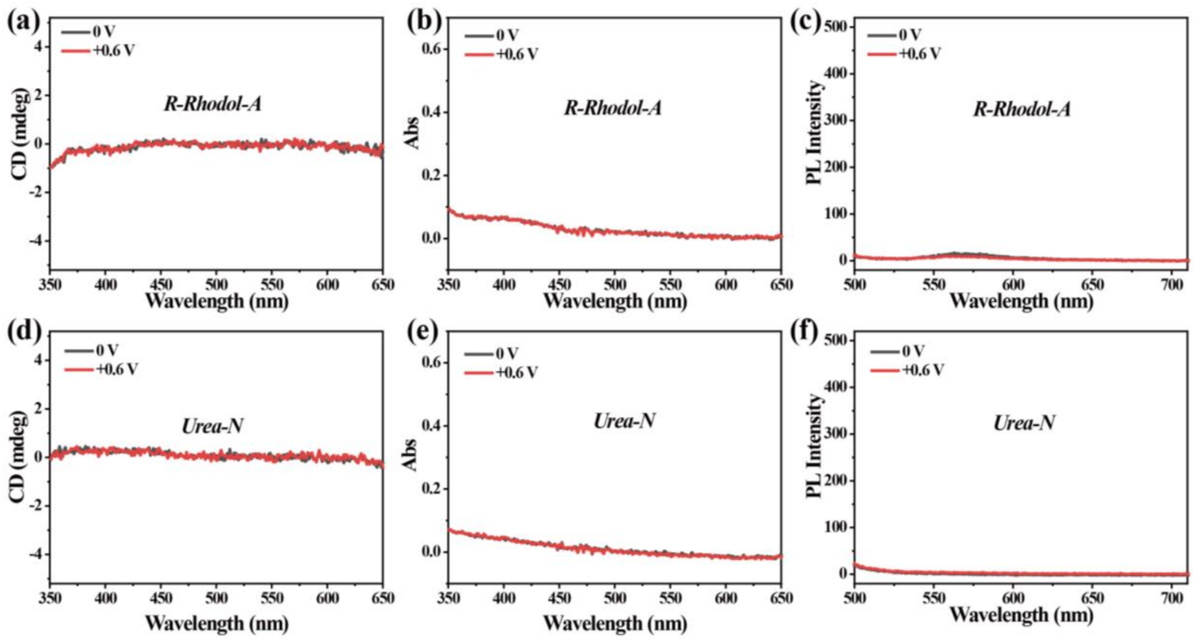

Figure S10. The *in situ* electrochemical (a) CD spectra, (b) UV-Vis absorption, and (c) fluorescence emission spectra (slit: 1.5, 3, ex: 365 nm) of 2.0×10^{-4} M *R-Rhodol-A* in acetonitrile with 1.0×10^{-1} M $TBAPF_6$. (d=1 mm). The *in situ* electrochemical (d) CD spectra, (e) UV-Vis absorption and (f) fluorescence emission spectra (slit: 1.5, 3, ex: 365 nm) of 1.0×10^{-3} M *Urea-N* in acetonitrile with 1.0×10^{-1} M $TBAPF_6$. (d=2 mm)

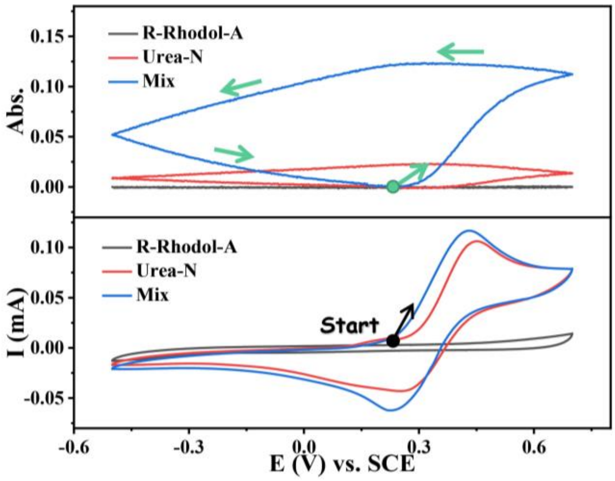

Figure S11. Changes in absorption at 532 nm (top) during CV (bottom) of 2.0×10^{-4} M *R-Rhodol-A* , 1.0×10^{-3} M *Urea-N* and the mixture (mix) of 2.0×10^{-4} M *R-Rhodol-A* and 1.0×10^{-3} M *Urea-N* in acetonitrile with 1.0×10^{-1} M $TBAPF_6$ at 20 mV·s^{-1}.

4. Supplementary Figures for devices

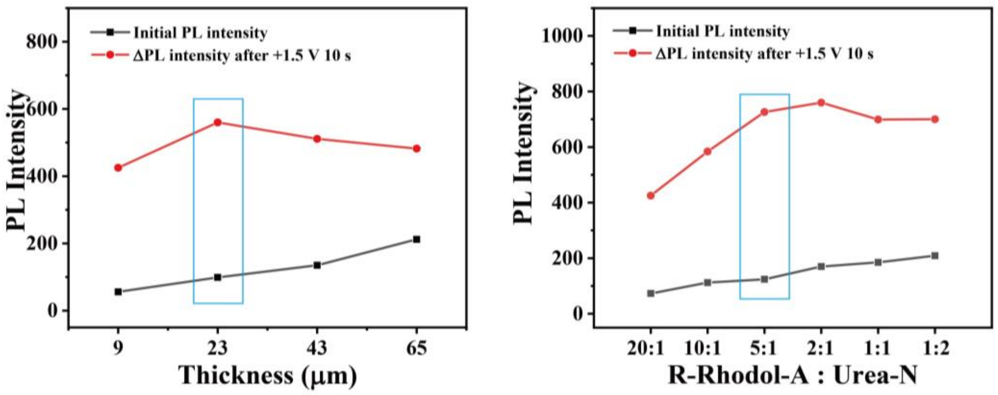

Figure S12. Optimization of fabrication parameters of the device. Initial PL intensity, and ΔPL intensity after application of +1.5 V 10 s for the devices with different (a) thickness of the CPL switching layer, (b) the molar ratio of *R-Rhodol-A* to *Urea-N* (slit: 3, 5, ex: 410 nm).

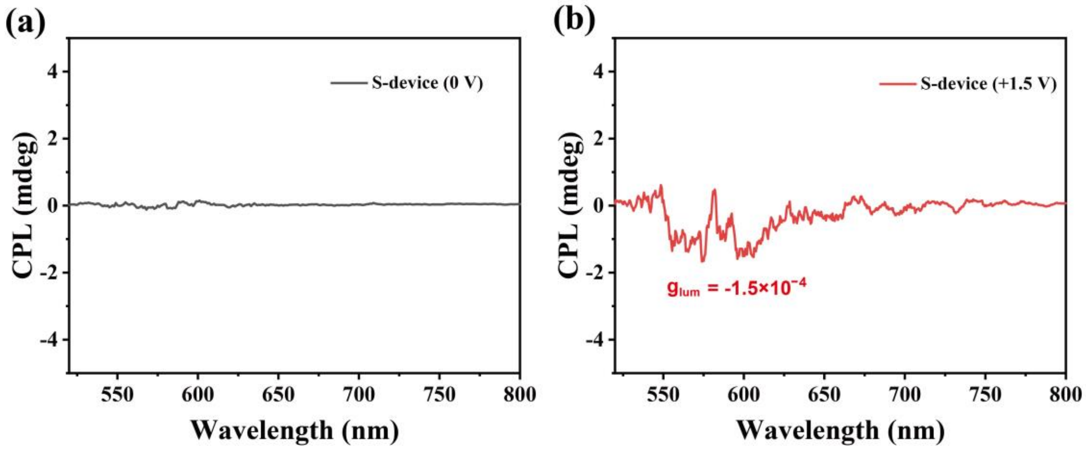

Figure S13. CPL spectra of the S-device before and after added suitable voltage (+1.5 V). (λ_{ex}=410 nm)

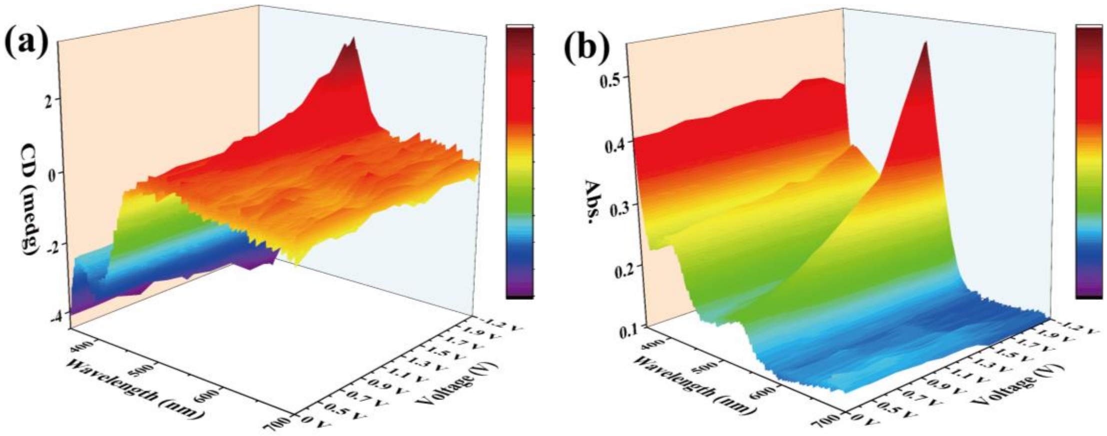

Figure S14. (a) CD and (b) UV-Vis absorption spectra of the device under various voltages.

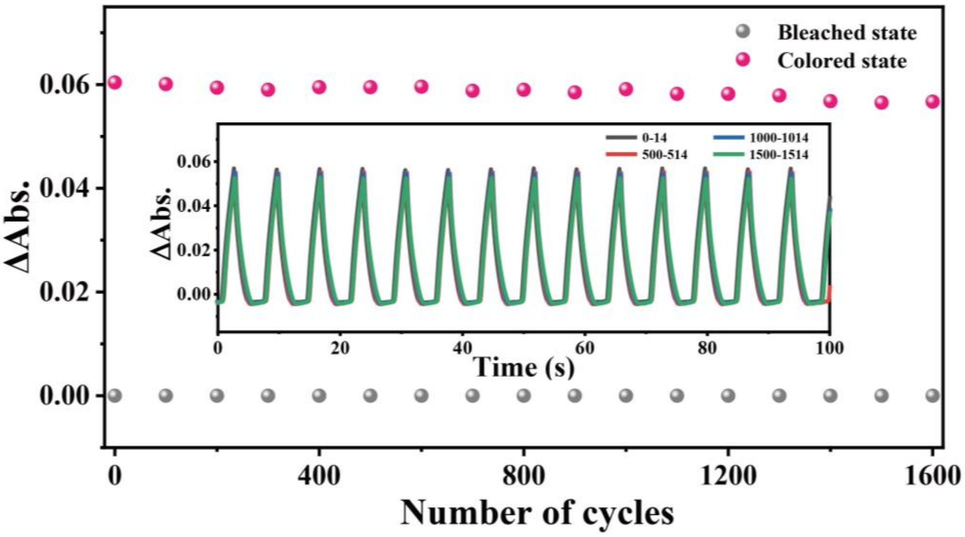

Figure S15. Cycling stability of absorption of the device under a suitable e-field. (Recorded at 574 nm, +1.5 V for coloring, -1.2 V for bleaching)

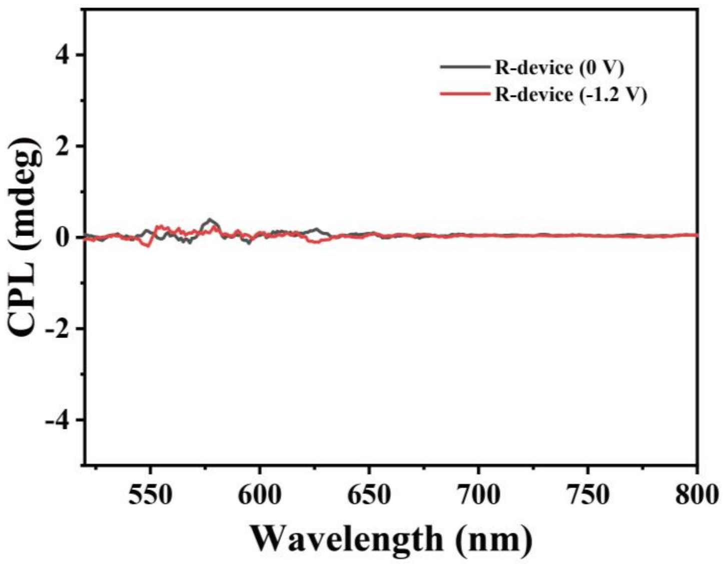

Figure S16. The CPL spectra of R-device at prepared “CPL-OFF” state (0V) and the recovered “CPL-OFF” state under -1.2 V after open circuit for 1 h, respectively. (λ_{ex}=410 nm)

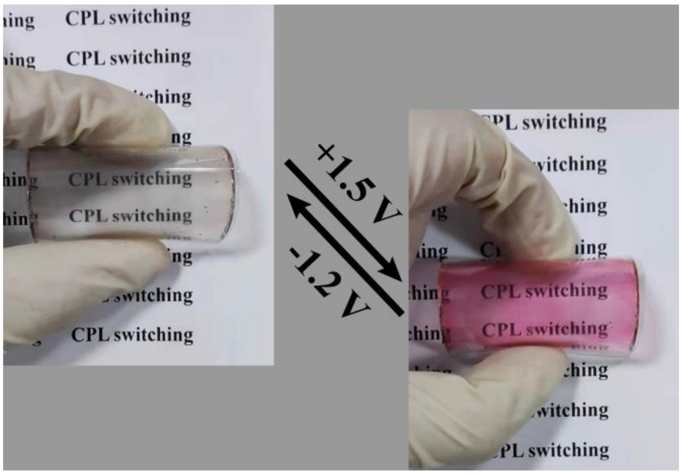

Figure S17. Physical display (reflective mode) of the flexible device.

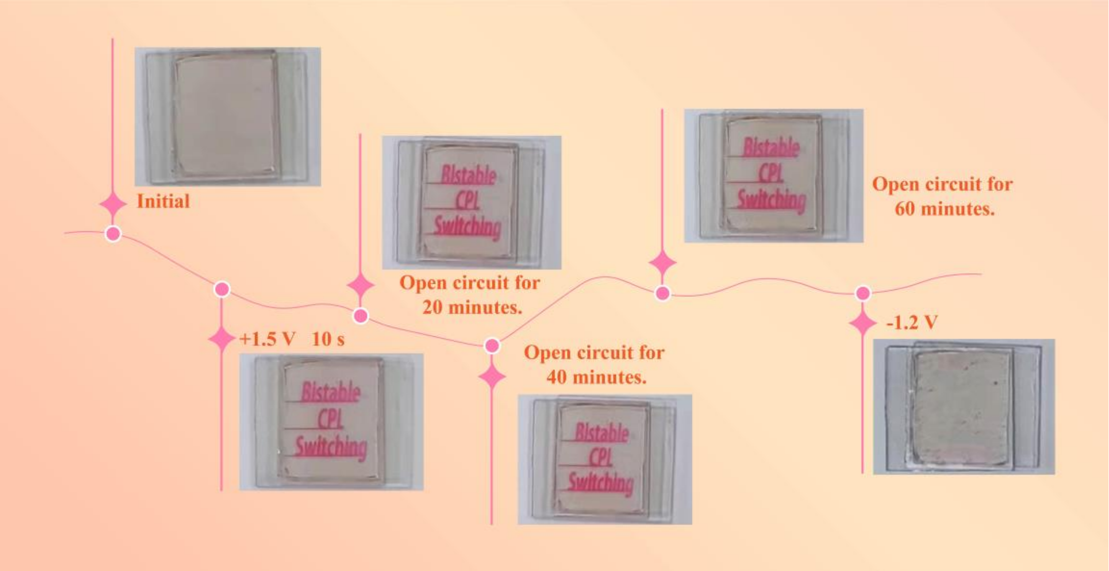

Figure S18. Detailed bistable color-switching process of the device (+1.5 V for coloring, then power off 3600 s, -1.2 V for bleaching).

5. ^{1}H and ^{13}C NMR spectra

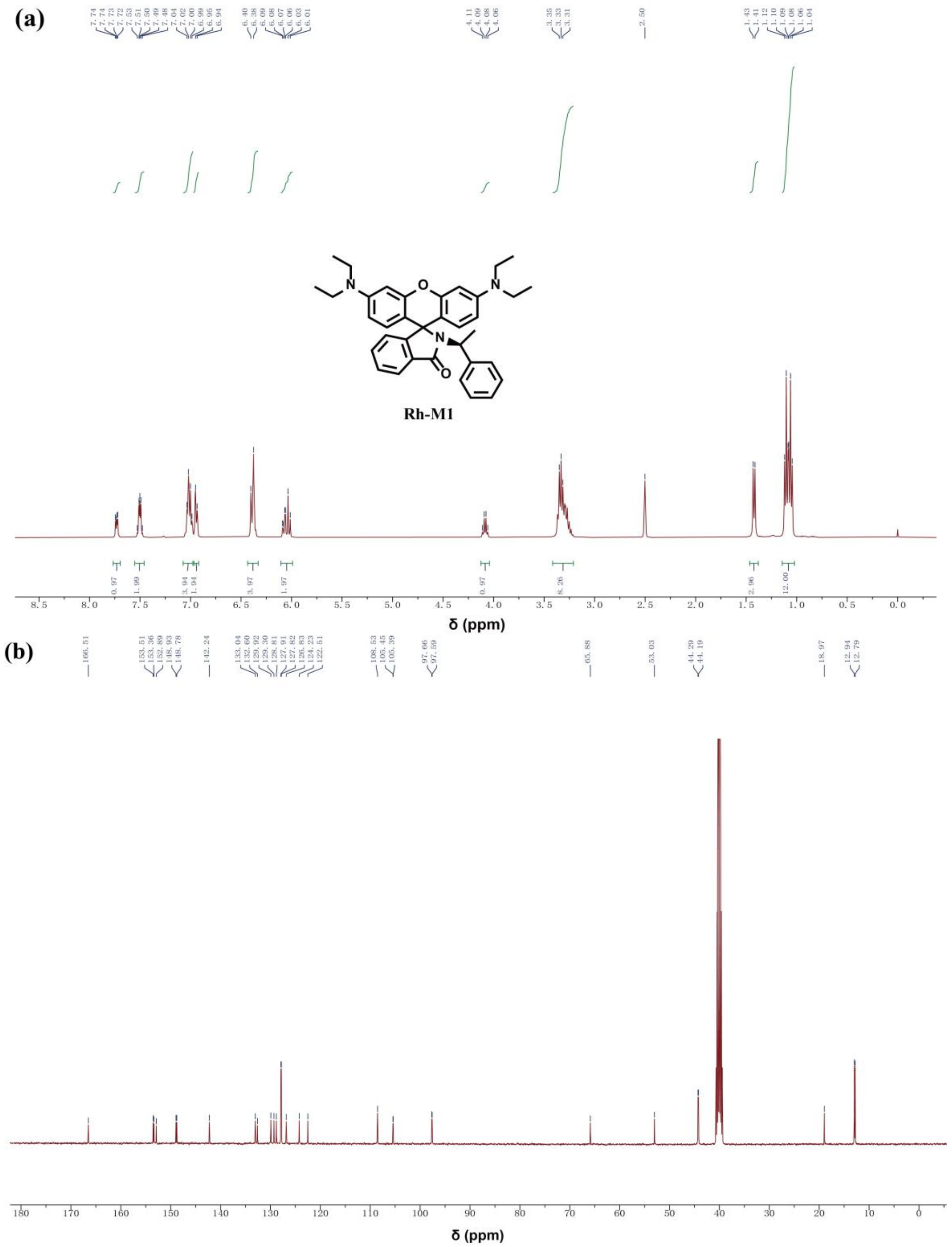

Figure S19. (a) ^{1}H NMR and (b) ^{13}C NMR spectra of *Rh-M1* in deuterated DMSO recorded at 400 MHz at room temperature.

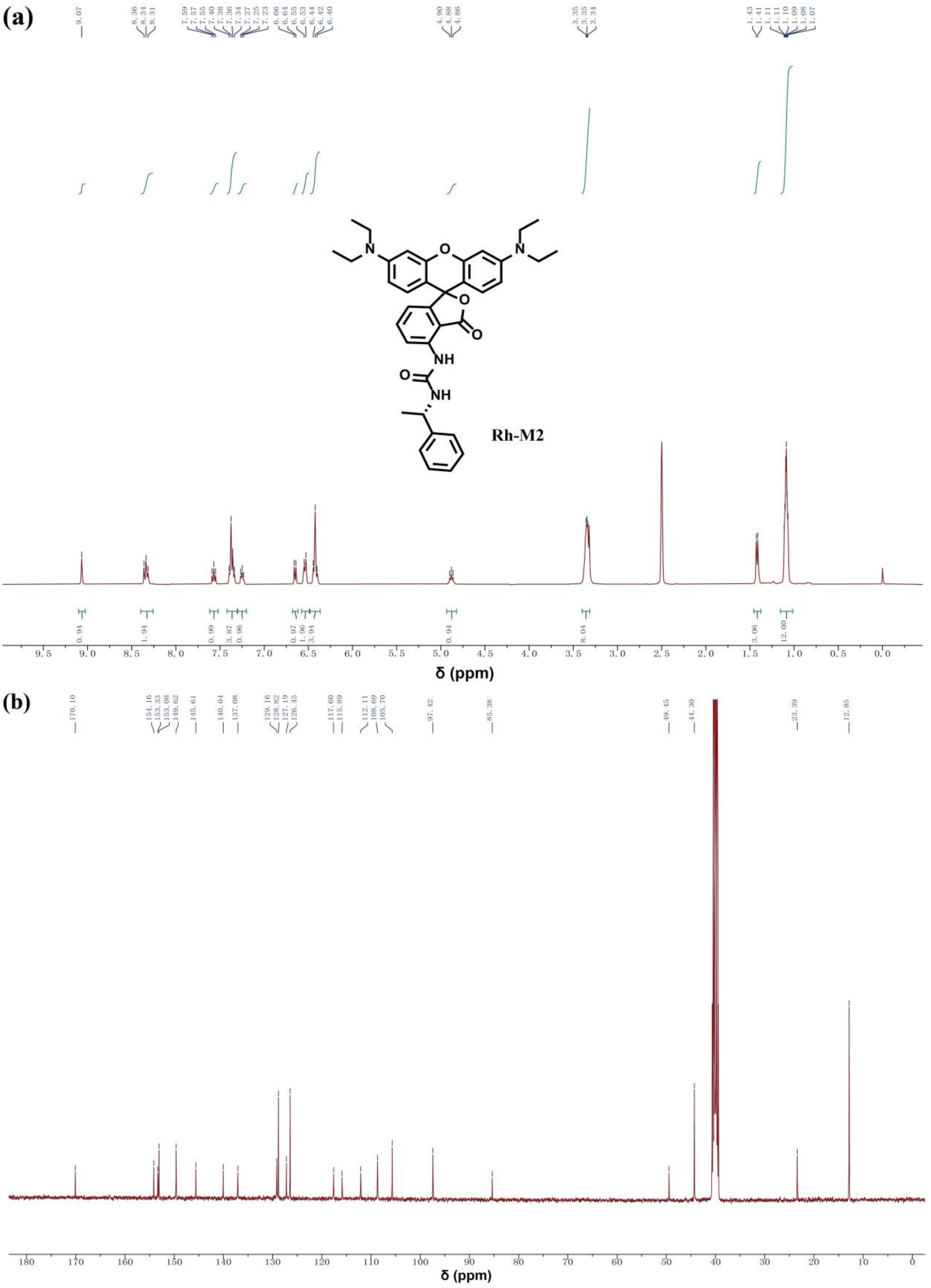

Figure S20. (a) ^{1}H NMR and (b) ^{13}C NMR spectra of *Rh-M2* in deuterated DMSO recorded at 400 MHz at room temperature.

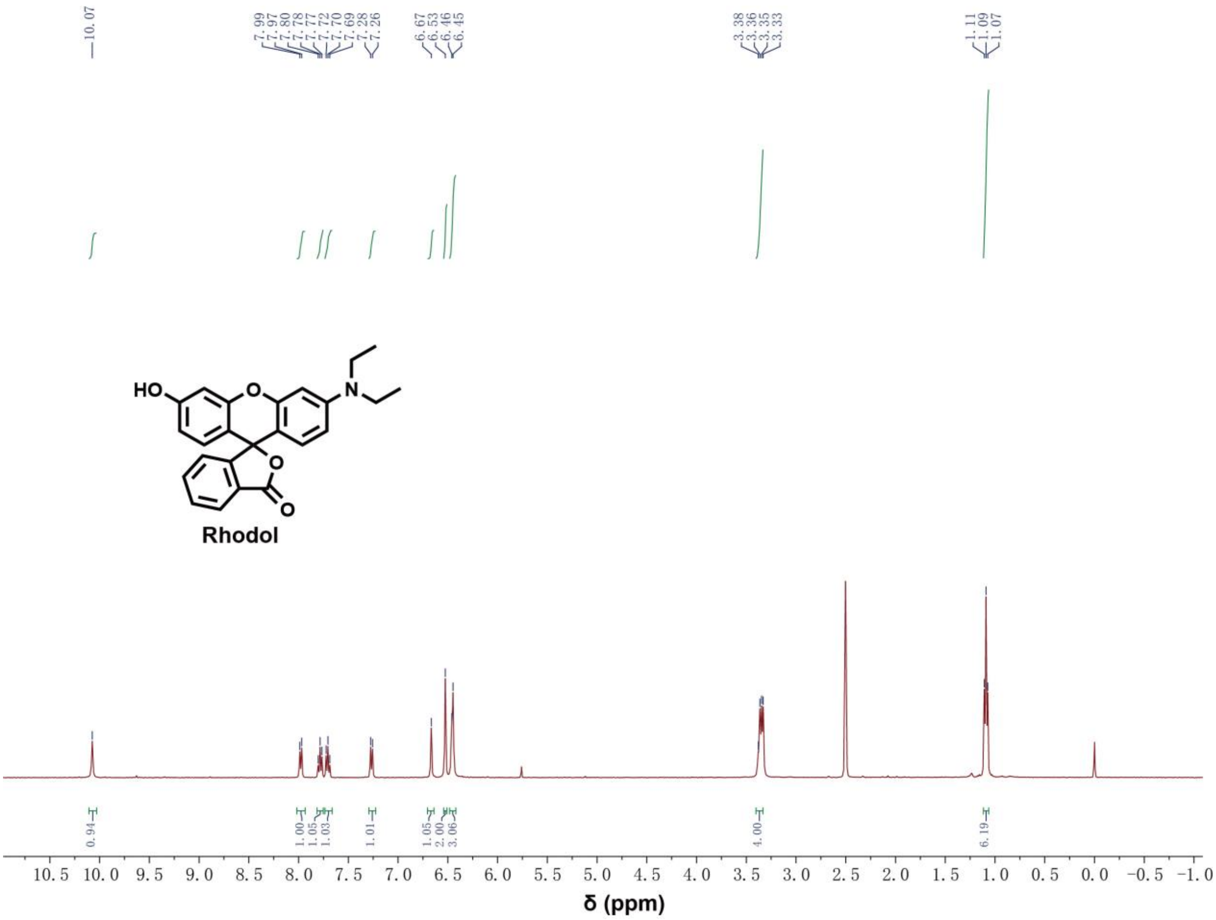

Figure S21. ^{1}H NMR spectra of *Rhodol* in deuterated DMSO recorded at 400 MHz at room temperature.

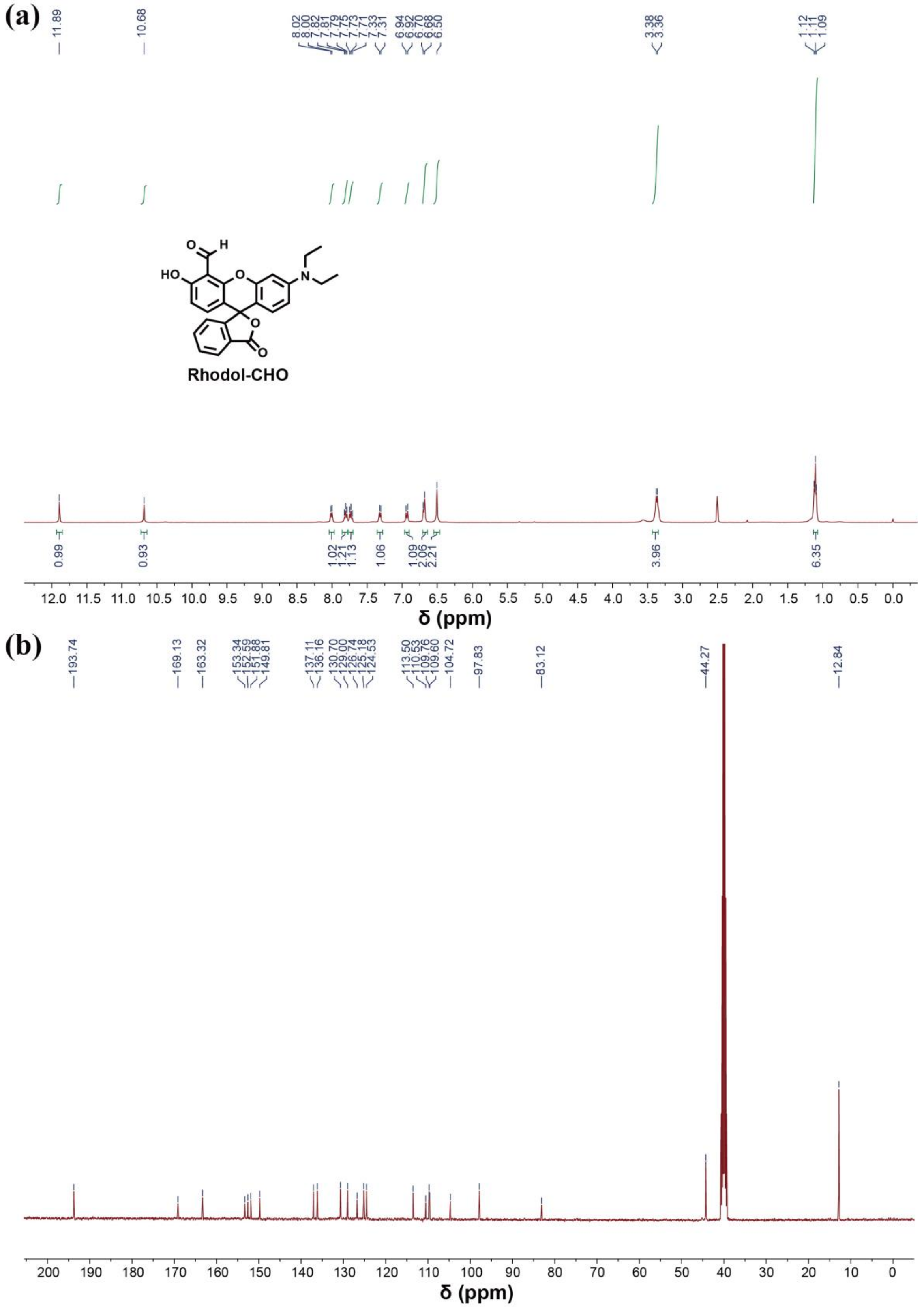

Figure S22. (a) ^{1}H NMR and (b) ^{13}C NMR spectra of *Rhodol-CHO* in deuterated DMSO recorded at 400 MHz at room temperature.

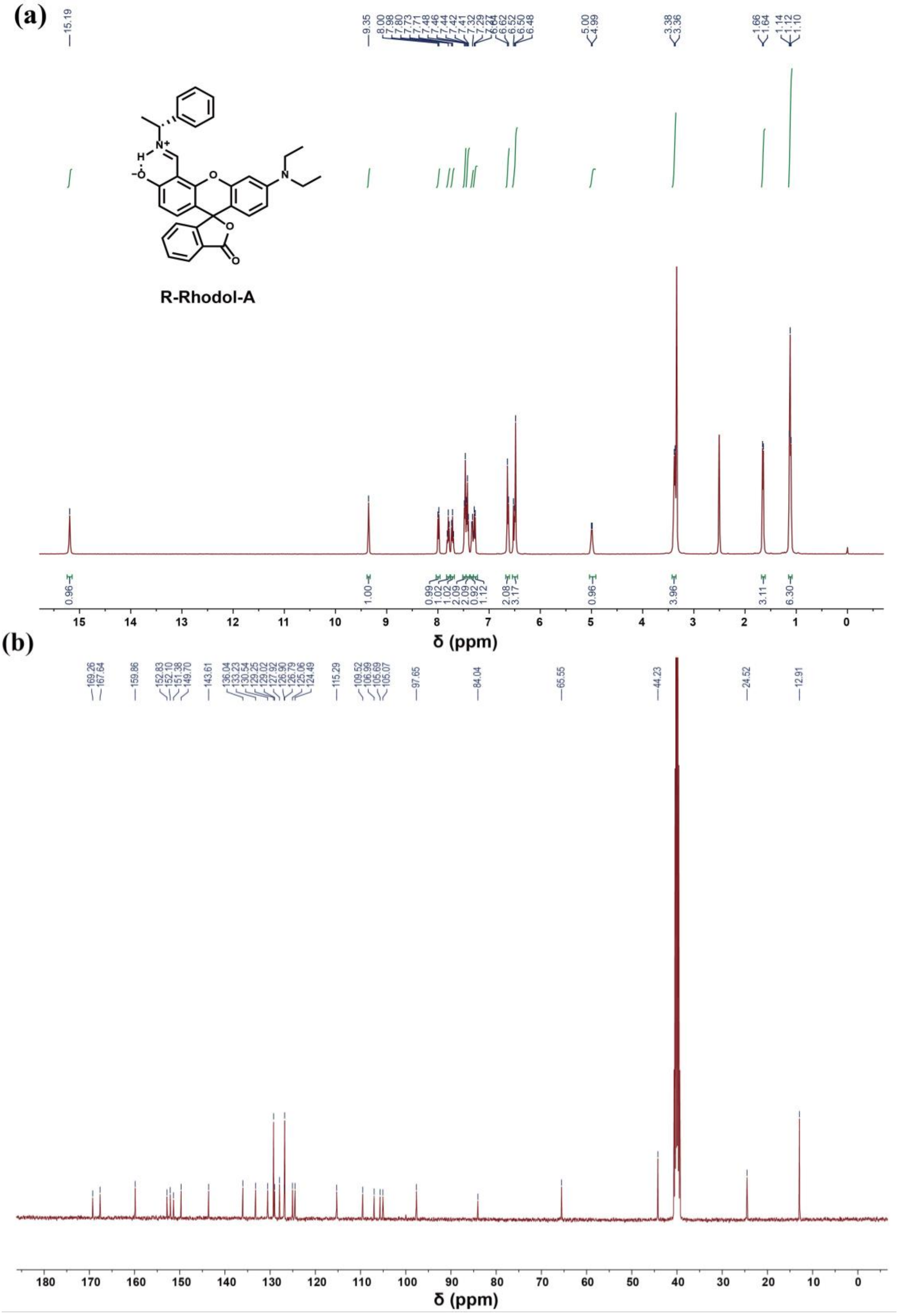

Figure S23. (a) ^{1}H NMR and (b) ^{13}C NMR spectra of *R-Rhodol-A* in deuterated DMSO recorded at 400 MHz at room temperature.

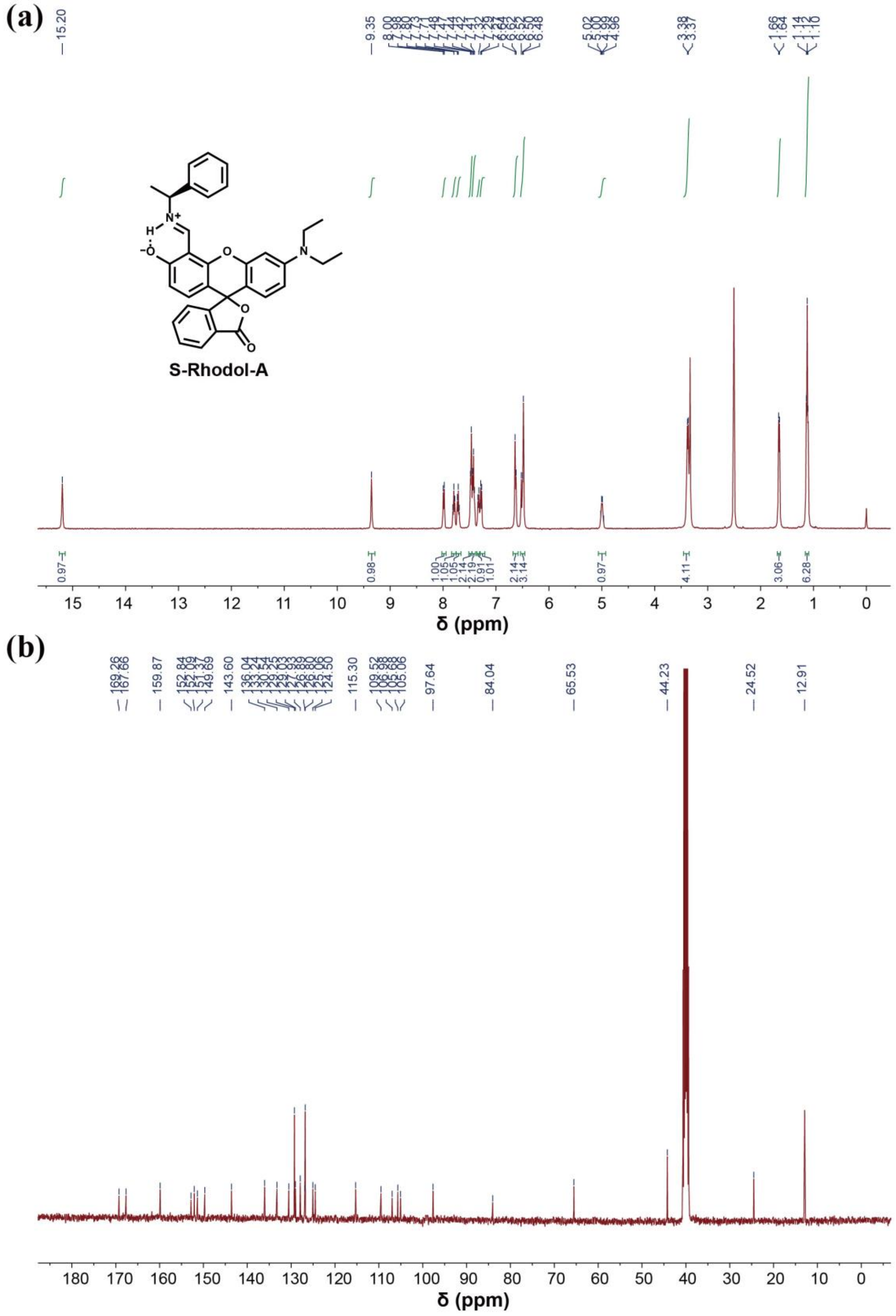

Figure S24. (a) ^{1}H NMR and (b) ^{13}C NMR spectra of *S-Rhodol-A* in deuterated DMSO recorded at 400 MHz at room temperature.

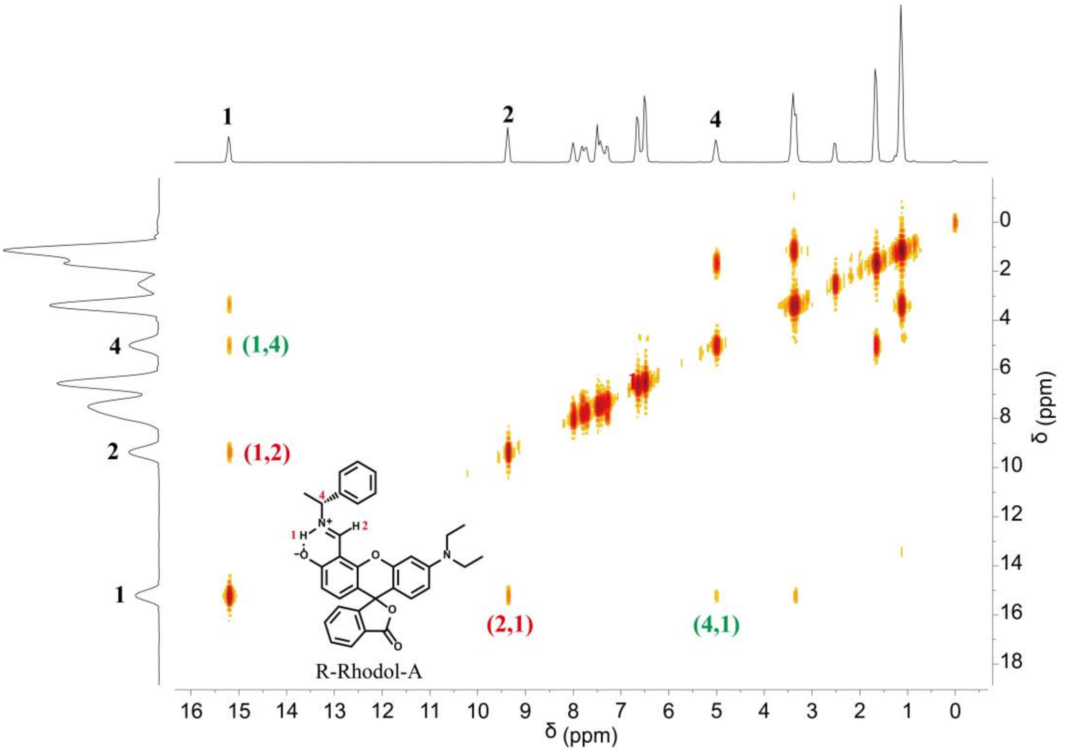

Figure S25. ^{1}H COSY spectrum of *R-Rhodol-A* in deuterated DMSO recorded at 400 M Hz at room temperature.

Discussion on the structure of *R-Rhodol-A*: The structure of *R-Rhodol-A* was determined by ^{1}H, ^{13}C, and ^{1}H COSY nuclear magnetic resonance (NMR) spectra (**Figure S24** and **S25**). The pattern of intramolecular hydrogen bond was ph-O^-•••HN^+R_3 rather than the ph-OH•••NR_3, according to the strong coupling between H1 and H4, as well as H1 and H2, which kept the same/similar with the structure of base-responsive chiroptical switch reported in our previous work (Angew. Chem. Int. Ed., 2021, 60, 2018-2023).

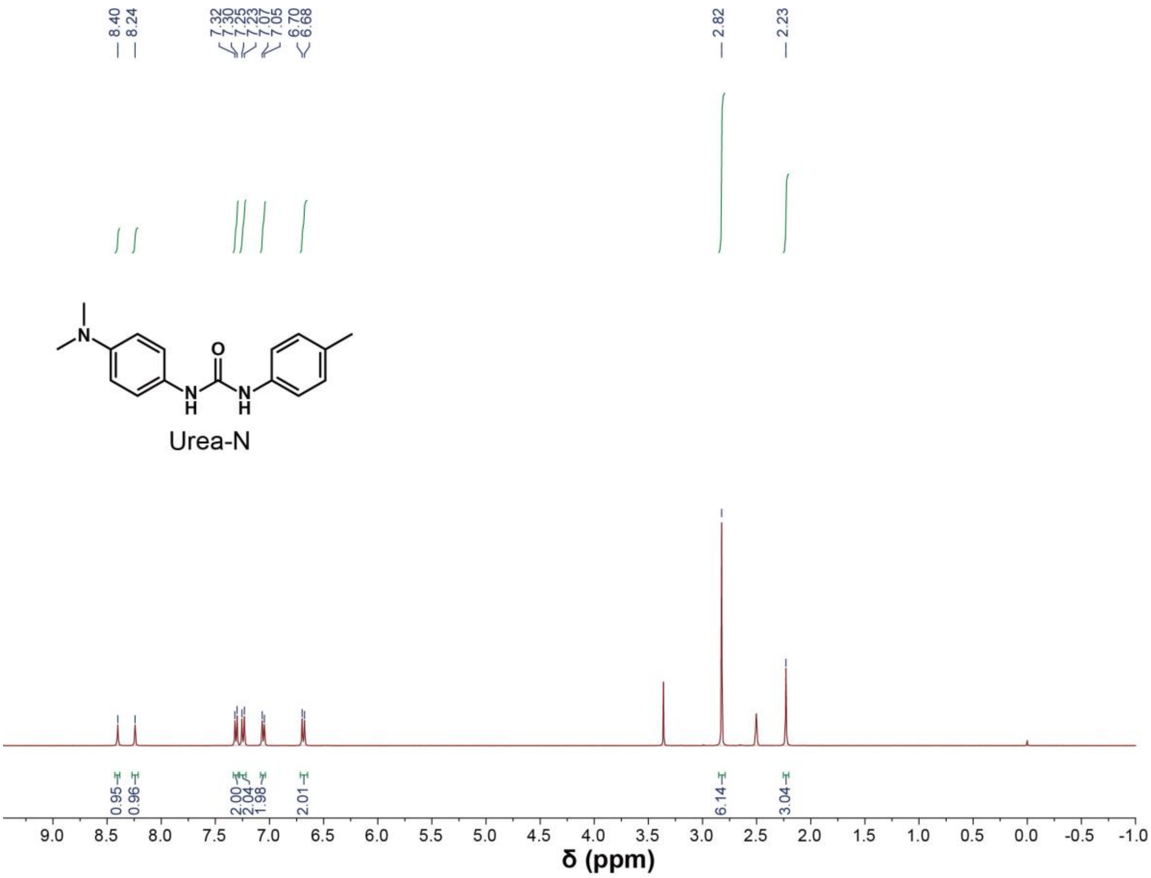

Figure S26. ^{1}H NMR spectra of *Urea-N* in deuterated DMSO recorded at 400 MHz at room temperature.

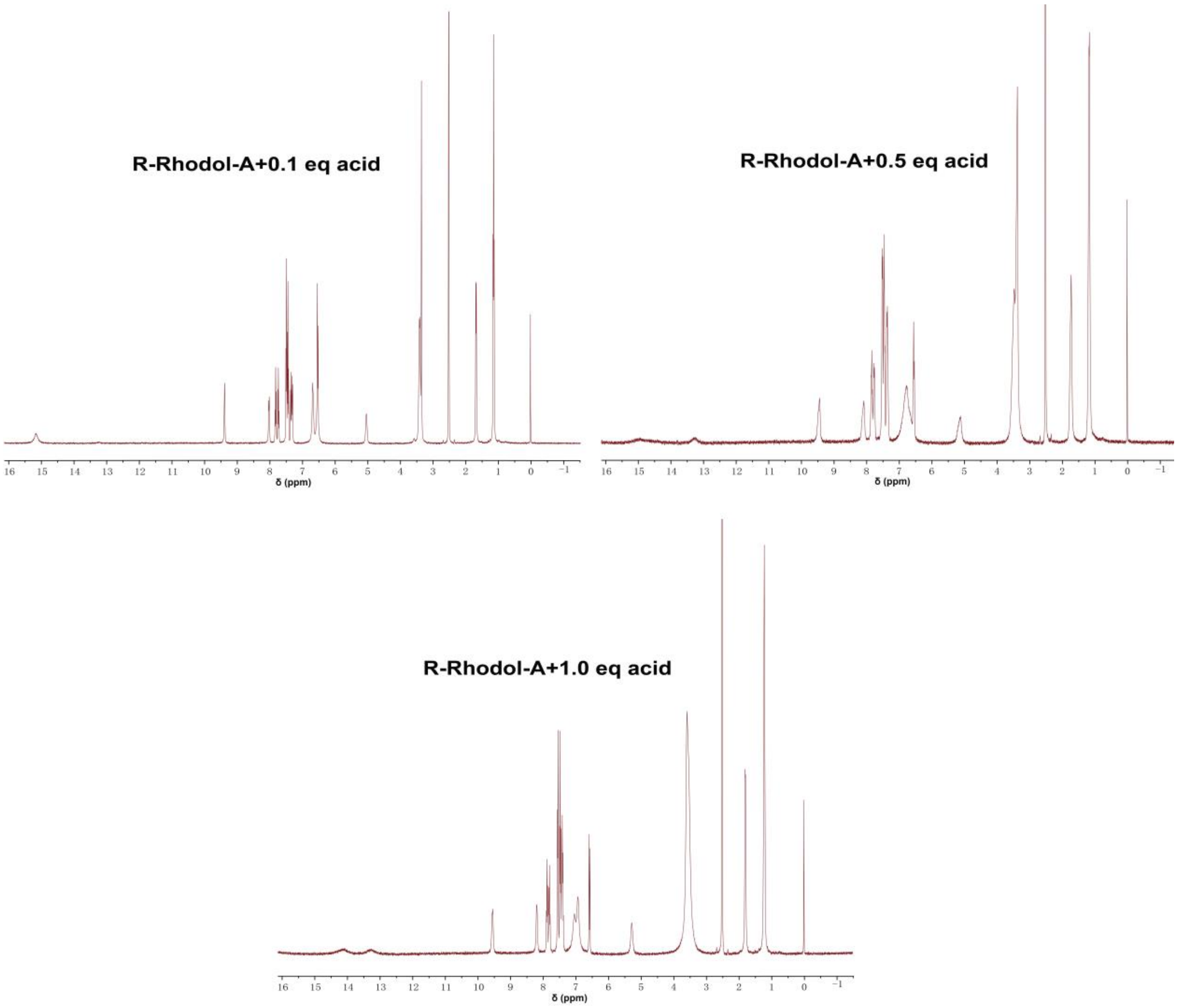

Figure S27. ^{1}H NMR spectra of 2.0×10^{-4} M *R-Rhodol-A* when added various eq CF_3COOH in deuterated DMSO recorded at 400 MHz at room temperature.

6. Comparison and References

Materials	Flexibility	Bistability	Cycling stability	$\lvert g_{lum}\rvert$	Ref. and year
Disubstituted polyacetylene & N-LCs*	*No*	*No*	*/*	*1.79*	[S1] *and 2014*
NO_2-CS-C_6-Chol & N-LCs*	*No*	*No*	*>4*	*0.38*	[S2] *and 2020*
D-A type chiral CPL emitters & N-LCs*	*No*	*No*	*>10*	*0.86*	[S3] *and 2020*
UCNPs & PKNCs & N-LCs*	*No*	*No*	*>5*	*1.1*	[S4] *and 2020*
Supramolecular gel system	*No*	*No*	*>10*	*/*	[S5] *and 2021*
Erovskite Confined in Lanthanide MOFs	*No*	*No*	*>7*	7.3×10^{-4}	[S6] *and 2022*
A-cyanodiarylethene-based molecule & N-LCs*	*No*	*No*	*>6*	*1.25*	[S7] *and 2022*
Urea-N & R-Rhodol-A	*Yes*	*1h*	*>1600*	3.5×10^{-4}	*This work*

Figure S28. Table of the summary of the reported representative CPL switching devices.

References

[S1] San Jose, B. A.; Yan, J.; Akagi, K. Angew. Chem. Int. Ed. **2014**, *53*, 10641-10644.

[S2] Chen, Y.; Lu, P.; Li, Z.; Yuan, Y.; Ye, Q.; Zhang, H. ACS Appl. Mater. Interfaces **2020**, *12*, 56604-56614.

[S3] Li, X.; Shen, Y.; Liu, K.; Quan, Y.; Cheng, Y. Mater. Chem. Front. **2020**, *4*, 2954-2961.

[S4] Yang, X.; Zhou, M.; Wang, Y.; Duan, P. Adv. Mater. **2020**, *32*, 2000820.

[S5] Du, S.; Zhu, X.; Zhang, L.; Liu, M. ACS Appl. Mater. Interfaces **2021**, *13*, 15501-15508.

[S6] Zhang, C.; Li, Z.-S.; Dong, X.-Y.; Niu, Y.-Y.; Zang, S.-Q. Adv. Mater. **2022**, *34*, 2109496.

[S7] Qiao, J.; He, Y.; Lin, S.; Fan, Q.; Guo, J. J. Mater. Chem. C **2022**, *10*, 7311-7318.
